# Identification of candidate genes associated with host-seeking behavior in the parasitoid wasp *Diachasmimorpha longicaudata*

**DOI:** 10.1186/s12864-024-10034-6

**Published:** 2024-02-06

**Authors:** Juan P. Wulff, Lucila M. Traverso, Jose M. Latorre-Estivalis, Diego F. Segura, Silvia B. Lanzavecchia

**Affiliations:** 1 Entomology and Plant Pathology, NCSU, Raleigh, NC USA; 2 Laboratorio de Neurobiología de Insectos (LNI), Centro Regional de Estudios Genómicos, Facultad de Ciencias Exactas, Universidad Nacional de La Plata, CENEXA, CONICET, La Plata, Bs As, Argentina; 3 Laboratorio de Insectos Sociales, Biología Molecular y Neurociencias (IFIBYNE), Instituto de Fisiología, Universidad de Buenos Aires - CONICET, Bs As, Buenos Aires, Argentina; 4 Laboratorio de Insectos de Importancia Agronómica, Instituto de Genética Ewald A. Favret (INTA) gv-IABIMO (CONICET), Hurlingham, Bs As, Argentina; 5 Facultad de Ciencias Agrarias y Veterinarias, Universidad del Salvador, Bs As, Argentina

**Keywords:** RNA-Seq, Differential gene expression (DGE) analysis, Sensory-related genes, Membrane receptor, Soluble receptor, Chemoreceptor, Mechanoreceptor, Female host-seeking behavior, Parasitoid wasp, Fruit fly parasitoid

## Abstract

**Background:**

*Diachasmimorpha longicaudata* is a hymenopteran fruit fly endoparasitoid. Females of this species find their hosts for oviposition by using complex sensorial mechanisms in response to physical and chemical stimuli associated with the host and host habitat. Ecological and behavioral aspects related to host-seeking behavior for oviposition have been extensively studied in *D. longicaudata*, including the identification of volatile organic compounds acting as attractants to females. In this sense, molecular mechanisms of chemoreception have been explored in this species, including a preliminary characterization of odorant-binding proteins (OBPs), chemosensory proteins (CSPs) and odorant receptors (ORs), among other proteins. Functional assays on OBP and CSP have been conducted as a first approach to identify molecular mechanisms associated with the female host-seeking behavior for oviposition. The aims of the present study were to identify the *D. longicaudata* sensory gene repertoire expressed in the antenna of sexually mature and mated individuals of both sexes, and subsequently, characterize transcripts differentially expressed in the antennae of females to identify candidate genes associated with the female host-seeking behavior for oviposition.

**Results:**

A total of 33,745 predicted protein-coding sequences were obtained from a *de novo* antennal transcriptome assembly. Ten sensory-related gene families were annotated as follows: 222 ORs, 44 ionotropic receptors (IRs), 25 gustatory receptors (GRs), 9 CSPs, 13 OBPs, 2 ammonium transporters (AMTs), 8 *pickpocket* (PPKs) receptors, 16 transient receptor potential (TRP) channels, 12 CD36/SNMPs and 3 Niemann-Pick type C2 like proteins (NPC2-like). The differential expression analysis revealed 237 and 151 transcripts up- and downregulated, respectively, between the female and male antennae. Ninety-seven differentially expressed transcripts corresponded to sensory-related genes including 88 transcripts being upregulated (87 ORs and one TRP) and nine downregulated (six ORs, two CSPs and one OBP) in females compared to males.

**Conclusions:**

The sensory gene repertoire of *D. longicaudata* was similar to that of other taxonomically related parasitoid wasps. We identified a high number of ORs upregulated in the female antenna. These results may indicate that this gene family has a central role in the chemoreception of sexually mature females during the search for hosts and host habitats for reproductive purposes.

**Supplementary Information:**

The online version contains supplementary material available at 10.1186/s12864-024-10034-6.

## Background

Hymenopteran parasitic wasps are considered the main natural enemies of several insect pest species of economic importance [1]. Female parasitoids find suitable hosts for oviposition guided by different stimuli, primarily chemical compounds [2, 3]. External chemical compounds are internalized through specific structures (sensilla), mainly located in the antennae, legs, and ovipositor [4–6]. Then, these chemical compounds are transported through the sensilla by odorant-molecule carriers to membrane-bound receptors located on the dendrites of olfactory sensory neurons (OSNs), which respond to the stimuli by triggering diverse biochemical pathways [7].

In insects, odorant (ORs) and gustatory (GRs) receptors are gene families coding for proteins constituted by seven transmembrane domains [7]. ORs respond to a variety of volatile organic compounds (VOCs), including pheromones and general odorants [8], whereas GRs sense non-volatile compounds by direct contact through gustatory sensilla but are also associated with carbon dioxide (CO_2_) detection [9]. Ionotropic receptors (IRs) are other membrane sensory-related genes associated with taste and olfaction [10]. Odorant-binding proteins (OBPs), chemosensory proteins (CSPs) and Niemann-Pick proteins type C2 (NPC2s) are carrier proteins highly expressed in the sensilla endolymph, associated with the transport of chemical compounds from the cuticle to the ORs, GRs and IRs, among other membrane-bound receptors located on the dendrites of OSNs [7]. 

Different chemosensory-related gene families have been studied in parasitoids species, namely, membrane receptors such as ORs, GRs and IRs [11–14], and carriers, including CSPs, OBPs and NPC2s [15–17]. By focusing on highly and differentially expressed genes in the female antennae, it has been possible to identify candidate chemosensory-related genes associated with female host-seeking behavior for oviposition in parasitoid species such as *Cotesia vestalis* Haliday (Hymenoptera: Braconidae) and *Microplitis mediator* Haliday (Hymenoptera: Braconidae) [14–16, 18, 19]. Functional studies, such as RNA interference, molecular docking and competitive fluorescence binding assays (including attractant VOCs for female parasitoids), have given further evidence of the role of specific ORs and OBPs in host detection in parasitoid wasps [20–25].

Other sensory-related gene families, which have not been studied in parasitoid species, could have a role in the female host-seeking behavior for oviposition. Ammonium transporters (AMTs) were recently suggested as insect non-canonical chemoreceptors [26]. These proteins have been identified in the antennae of different insect species [27, 28]. Positive AMT staining was found in olfactory neurons in the antenna of *Drosophila melanogaster* Meigen (Diptera: Drosophilidae) [28], and functional studies on the same species have given further evidence about their role as odorant receptors [28]. *Pickpocket* (PPK) receptors were found to be involved in feeding [29, 30] and pheromone detection [31] in *D. melanogaster*. PPK transcript expression was observed in OSNs co-expressing with IR84a, a receptor associated with food-derived odor sensation [32]. Further, among other sensory-related gene families, members of the transient receptor potential (TRP) gene family have been found to be expressed in the antenna of many insect species [27, 33, 34]. In *D. melanogaster*, the TRPC sub-family was associated with CO_2_ sensing, and an interaction with gustatory receptors *DmelGr63a* and *DmelGr21a* was suggested [35]. TRPA and TRPL sub-families have been associated with gustatory chemosensing in the same species [36, 37]. Regarding other gene families, members of the CD36 gene family located in OSNs participate in the olfactory signal transduction by the transport of lipophilic compounds [38, 39]. The sensory neuron membrane protein 1 (SNMP 1) is the most widely studied in insects [40]. In *D. melanogaster*, SNMP 1 acts as a co-receptor, assisting OBPs and ORs in sensing pheromone molecules, such as cis-vaccenyl acetate (cVA) [41]. Other members of the CD36, such as Scavenger Receptor Class B Type I and Croquemort proteins, have been identified in the insect antenna, with potential roles in chemoreception [42, 43].

The present study focuses on the parasitoid *Diachasmimorpha longicaudata* Ashmead (Hymenoptera: Braconidae). This species is an endoparasitoid considered the main biological control agent of tephritid fruit flies of economic importance [44]. Ecological and behavioral aspects related to host-seeking behavior for oviposition in *D. longicaudata* have been extensively studied [45, 46]. Further, specific VOCs have been identified to act as attractants to females [3]. However, a deeper understanding of the sensory-related gene repertoire of this parasitoid wasp is still necessary; only ORs, OBPs and CSPs, among other proteins, have been identified for this species [25, 47]. Further, functional assays to identify receptors associated with host-seeking behavior for oviposition have been conducted only for a few OBPs and CSPs [25]. 

The aims of the present study were (1) to identify the *D. longicaudata* sensory gene repertoire expressed in the antenna of sexually mature and mated females and males, including gene families not previously addressed in parasitoid wasps; (2) to characterize sensory-related transcripts differentially expressed in the antennae of sexually mature females to identify candidate genes associated with female host-seeking behavior for oviposition. The information obtained about putative molecular mechanisms of host detection in the female parasitoid may contribute to improve mass-reared strains through a directional selection of females with a higher performance in host-seeking behavior assisted by molecular techniques. 

## Results

### RNA-sequencing, ***de novo*** assembly and prediction of coding sequences

Sequencing of 10 cDNA libraries (five male + five female) yielded a total of 194,829,029 pair-end raw reads. The trimmed databases constituted a total of 95,032,084 and 89,684,457 reads for female and male libraries, respectively (Table S1: Sheet A). The latter were used to generate a *de novo* assembly that contained 163,327 transcripts and a guanine-cytosine (GC) content of 40.34% (Table S1: Sheet B). The range of successfully mapped reads against our non-redundant predicted protein-coding sequence (CDS) database was between 8,476,678 and 10,021,469 per library (Table S1: Sheet A). After removing redundancy 33,745 CDS were identified, and the Benchmarking Universal Single-Copy Orthologs (BUSCO) searches revealed more than 90% of complete BUSCOs in our non-redundant translated CDS database (Figure S1). 

### Phylogenetic analysis, transcript expression and differential expression analysis of sensory-related gene families

Analysis of the generated antennal CDS database allowed the identification and characterization of 10 sensory-related gene families. These gene families were manually curated and annotated as follows (number of transcripts with complete sequences are shown between parenthesis): ORs (204), IRs (44), GRs (22), CSPs (9), OBPs (12), AMTs (2), PPKs (8), TRPs (16), CD36/SNMPs (12) and NPC2-like (3). In addition, 18 ORs, three GRs and one OBPs with partial sequences were identified and annotated (Data File S1). BLAST searches against Insecta NCBI database showed the best hits vs. *Diachasma alloeum* Muesebeck (Hymenoptera: Braconidae) sequences, followed by *Fopius arisanus* Sonan (Hymenoptera: Braconidae), as detailed in (Table S2: Sheets A-J).

**Fig. 1:**
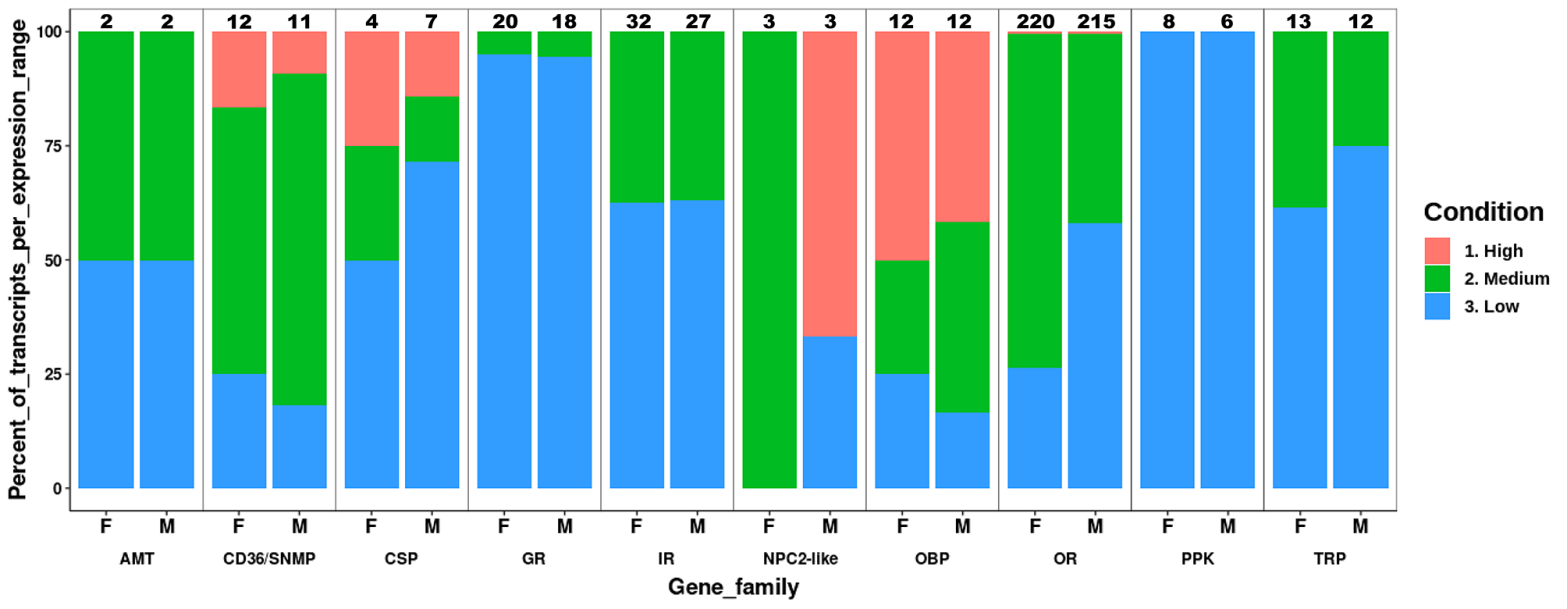
Percent of sensory-related transcripts per expression range. Only transcripts considered expressed in the antenna of *D. longicaudata* (TMP > 0.5) were plotted. The percent of transcripts (for each sensory-related gene family) per expression range (low, medium, high) was calculated on the total transcripts expressed in the *D. longicaudata* antenna (either female or male), detailed at the top of each column. AMT: ammonium transporter receptor, CD36/SNMP: CD36 and sensory neuron membrane protein family, CSP: chemosensory protein, GR: gustatory receptor, IR: ionotropic receptor, NPC2-like: Niemann-Pick C2 disease like protein, OBP: odorant-binding protein, OR: odorant receptor, PPK: *pickpocket* receptor, TRP: transient potential receptor channel, F: female antenna, M: male antenna

Based on the phylogenetic analysis of each gene family (Figure S2), all *D. longicaudata* sensory-related sequences were renamed as detailed in Table S3: Sheets A-J. Transcripts expression for the 10 sensory-related gene families were summarized in Fig. 1. Phylogenetic and transcript expression (provided in transcripts per million; TPM) results are further detailed below under each sensory-related gene family sub-section. For additional details refer to Table S1: Sheets D and E.

**Fig. 2:**
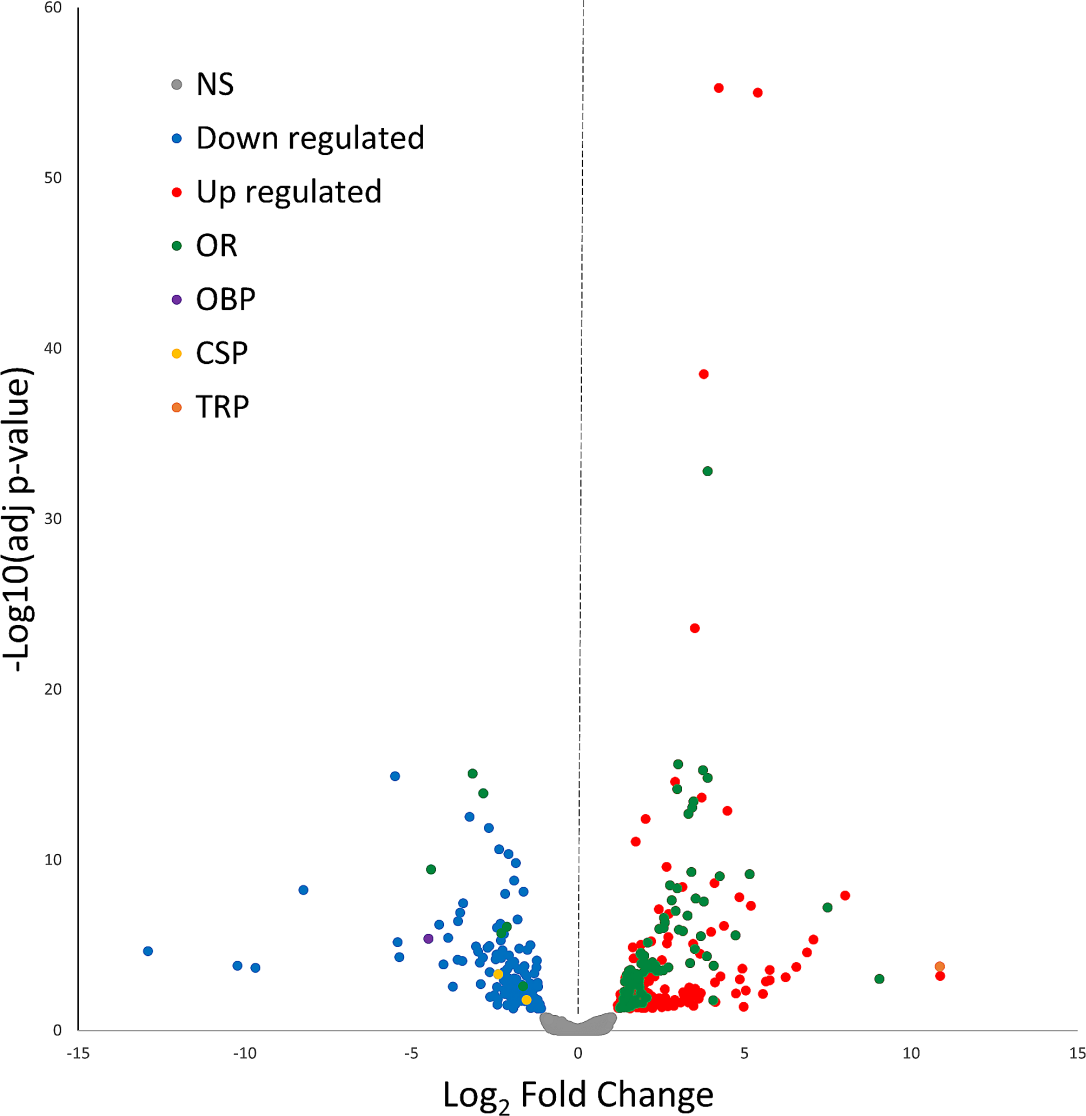
Volcano plot summarizing the differentially expressed sequences in female vs. male antennae of *D. longicaudata*. Transcript abundance is represented as Log_10_ (TPM + 1). The x-axis and y-axis represent Log_2_ fold-change (Log_2_FC) differences between the contrasted groups and statistical significance as the negative Log_10_ of adjusted *P*-value (s-value), respectively. A total of 13,342 genes were included in the analysis, yielding 237 and 151 transcripts, up- and downregulated in the female antenna vs. male antenna. Transcripts upregulated (Log_2_FC > 1 and s-value < 0.05) are indicated with red dots, those downregulated (Log_2_FC < 1 and s-value < 0.05) are highlighted in blue, and non-significant transcripts are shown as gray dots (*n* = 5 biological replicates per group). In addition, sensory-related genes that were differentially expressed between groups are highlighted as follows: odorant receptors (OR) in green, odorant-binding proteins (OBP) in purple, chemosensory proteins (CSP) in yellow, and transient receptor potential channels (TRP) in orange. Log_2_FC < 1 and s-value < 0.05 for all transcripts analyzed are detailed in Table S4: Sheets A and B

Highly variable and/or low expressed transcripts were removed from the 33,745 previously identified CDSs, resulting in 13,442 sequences used in differential gene expression (DGE) analysis. The DGE analysis summarized in a volcano plot (Fig. 2), revealed 237 and 151 transcripts up- and downregulated, respectively (Log_2_ Fold-Change (Log_2_FC) threshold > 1 and s-value < 0.05) in the female vs. male antennae. For additional details refer to Figure S3, a heatmap including all differentially expressed (DE) sequences between the female and male antenna. All sequences analyzed in the DGE analysis are provided in Table S4: Sheet A. In addition, BLAST searches for DE transcripts non-sensory related, are provided in Table S4: Sheet B.

**Fig. 3:**
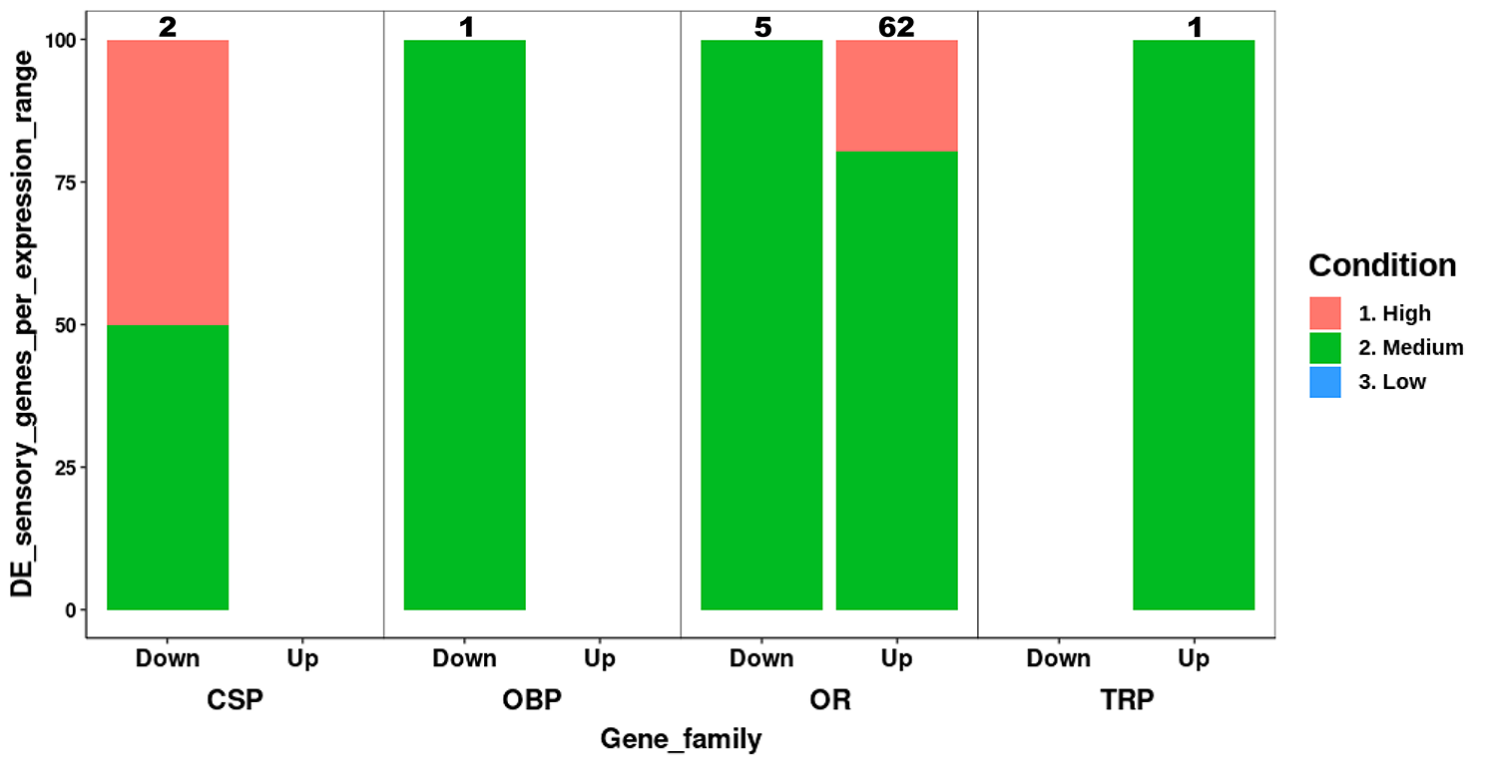
Percent of differentially expressed (DE) sensory-related genes per expression range. DE sensory-related genes for each gene family were plotted according to gene expression range (low, medium, high) and differential expression between sexes. The percent of genes per expression range was calculated on the total genes differentially expressed in the *D. longicaudata* antenna (either up or down regulated in the female antenna), detailed at the top of each column. CSP: chemosensory protein, OBP: odorant-binding protein, OR: odorant receptor, TRP: transient potential receptor channel, Down: downregulated in the female antenna, Up: upregulated in the female antenna

Ninety-seven DE transcripts (25% of total DE sequences) corresponded to sensory-related genes, with 88 transcripts being upregulated (37% of total up) and 9 downregulated (6% of total down) in females compared to males (Table S4: Sheet A). The 88 upregulated transcripts were distributed among the sensory-related gene families as follows: 66 ORs, 21 partial ORs (receptors presenting a length sequence shorter than 70% of their potential orthologs) and one TRP, sub-type transient receptor potential-like (TRPL), belonging to the TRPC subfamily. After manual curation of the DE transcripts, eight of the 66 upregulated ORs were reassigned as partial fragments forming four complete sequences, totalizing 62 upregulated ORs (Table S4: Sheet A). The downregulated transcripts in female antennae were distributed among the sensory-related gene families as follows: six ORs, two CSPs and one OBP. Considering that two of the six ORs were partial fragments of the same ORs, the final number of downregulated ORs in females was five (Table S4: Sheet A). The expression range (low, medium, high) for the manually curated DE sensory-related genes was summarized in Fig. 3.

Further, among other DE transcripts between the antenna of both sexes, 30 transcripts belonging to gene families potentially involved in odorant-molecule degradation were identified as follows: 14 cytochromes P450 (CYPs), 12 carboxylesterases (COesterases), three juvenile hormone acid O-methyltransferase (JHAMT) and one neprilysin protein (Table S4: Sheet A). Eight CYPs, one COesterase, three JHAMTs and one neprilysin were upregulated in the female antenna, and six CYPs and 11 COesterases were downregulated in the same tissue (Table S4: Sheet A).

#### Sensory-related membrane receptors

##### Odorant receptors

**Fig. 4:**
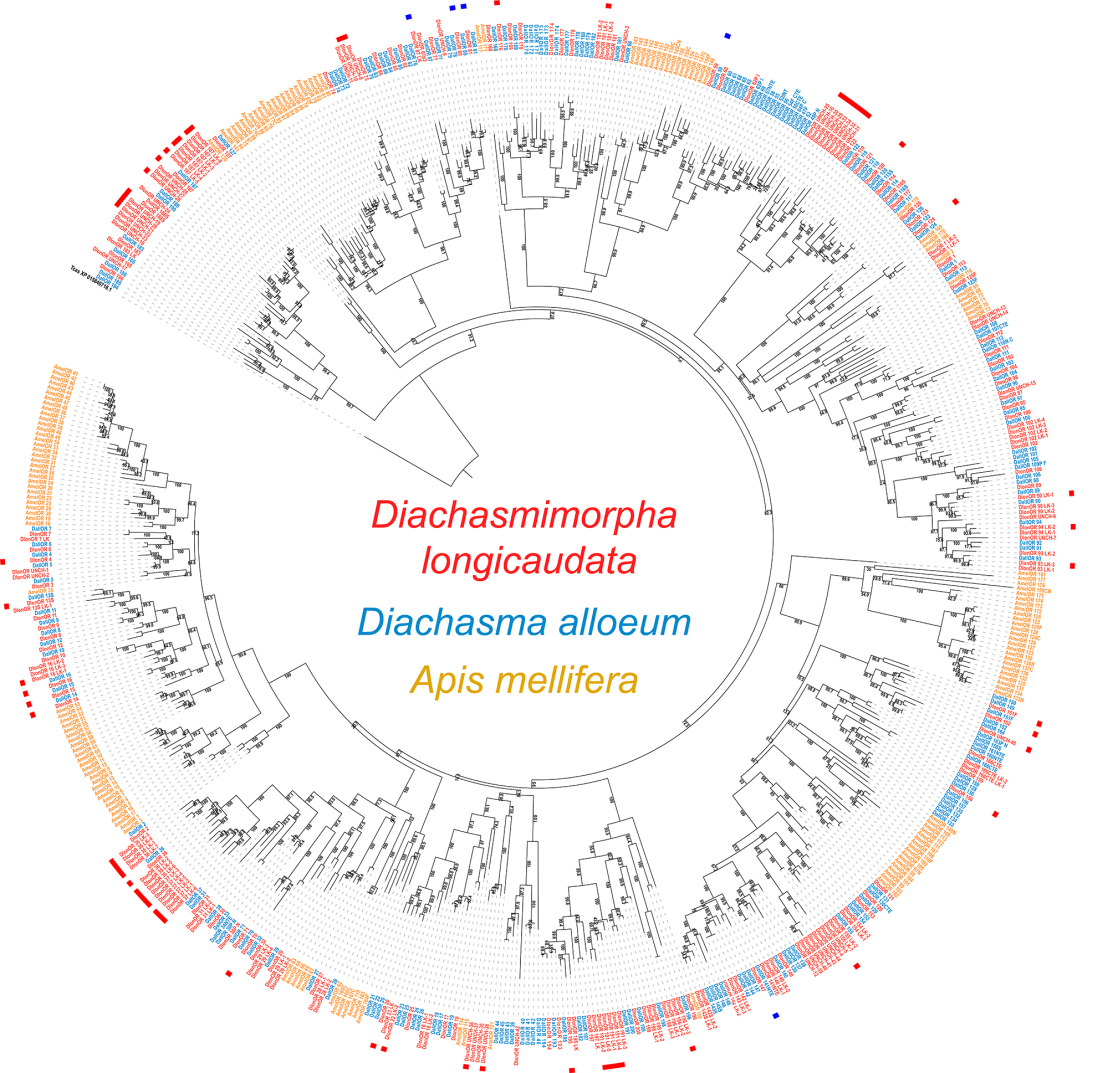
Phylogenetic analysis of odorant receptors from *D. longicaudata*, *D. alloeum* and *A. mellifera.* Three hymenopteran species were used for the phylogenetic analysis as follows: *D. longicaudata* (Dlon, red), *D. alloeum* (Dall, blue), *A. mellifera* (Amel, yellow). *T. castaneum* (Coleoptera) was used as an outgroup (Tcas, black). Odorant receptors up- and downregulated in the female antenna were differentiated with a red and blue tag, respectively. Numbers represent bootstrap values supporting each node resolution. OR: odorant receptor, LK: like, UNCH: uncharacterized

Clade expansions were identified for several ORs based on the OR phylogenetic analysis as detailed in Fig. 4. The upregulated ORs in the female antenna were distributed into 28 clades, six of which included three or more upregulated sequences (Fig. 4).

**Fig. 5:**
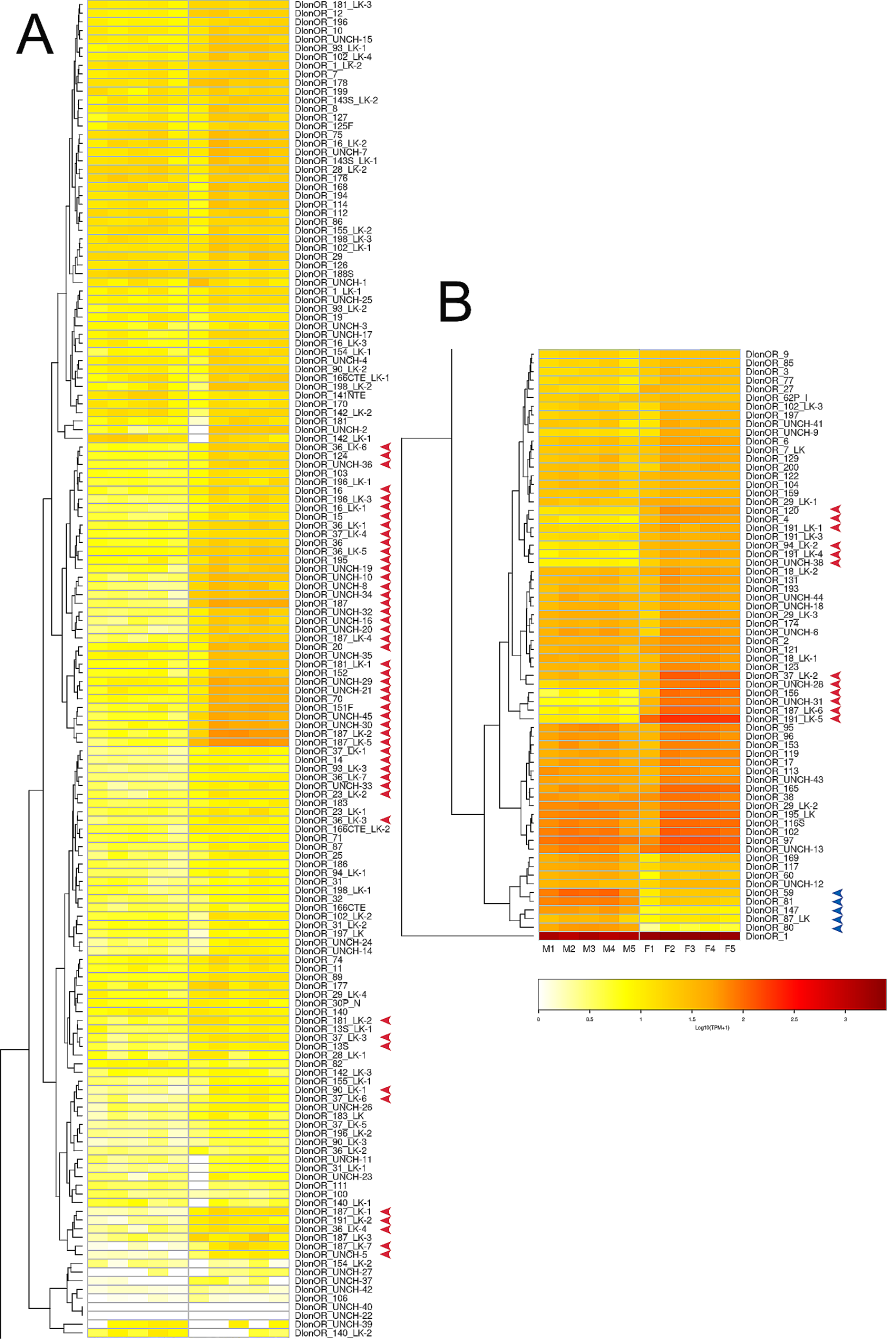
Antennal transcript abundance of odorant receptors in females and males *of D. longicaudata*. Transcript abundance was represented as Log_10_ (TPM + 1), and white and red colors represent the change from lowest to highest expression among libraries of the antennae of both sexes (*n* = 5 per group). Note that figure segment **(B)** continues from the bottom of segment **(A)**, since these segments were separated for better visualization of sequence IDs. A dendrogram was plotted using hierarchical clustering of gene expression values based on Euclidean distance and a complete linkage method for clustering. Receptors up- and downregulated in the female antenna vs. male antenna were highlighted with a red and blue arrow, respectively. Raw counts, TMP and Log_10_ (TPM + 1) values per sequence and library are presented in Table S1: Sheet D. OR: odorant receptor, TPM: transcript per kilobase per million, MA: male antenna, FE: female antenna, LK: like, UNCH: uncharacterized

The TPM value for each OR per library is provided in Fig. 5 and detailed information is provided in Table S1: Sheets D and E. Differential expressed ORs between female and male antenna were highlighted in the same figure and Supplementary Tables S1 and S4. Twenty of the 62 upregulated complete ORs in the female antenna showed medium expression (TPM > 10) (Fig. 1; Table S1: Sheets D and E) and the OR1 (odorant receptor co-receptor, Orco) was the only OR showing a high expression level (TMP > 1000) in both, female and male antennae (Fig. 1; Table S1: Sheets D and E).

Of the 83 OR transcripts upregulated in the female antenna (62 complete ORs after manual curation, plus 21 OR partial sequences), 46 of them showed a Log_2_FC ≥ 2, which means that these sequences were highly DE (≥ 200% increase) between the female and the male antenna (Fig. 2; Table S4: Sheet A). In addition, 25 of the 62 manually curated and upregulated ORs in the female antenna, showed medium expression (TPM > 10) plus > 200% increase in differential expression between females and males (Fig. 3). Further, 20 of those 25 ORs upregulated in the female antenna were grouped into six clades (C1-6) as follows: C1 determined by ORs 187 plus 187 LK1-7, includes six upregulated ORs, all of them showed an increase > 200%; C2 closely located to C1, included the uncharacterized (UNCH) ORs 19–23, three of them were upregulated plus two with an increase > 200%; C3 grouped UNCH-ORs 16 and 29–34, all members of this group were upregulated in the female antenna and five of them showed > 200% increase; C4 included OR 36 plus 36 LK1-7, all but one were upregulated plus one with an increase of > 200%; C5 was represented by ORs 191 LK1-5, four of them were upregulated and two with an increase > 200%; and C6 grouped UNCH-ORs 8 and 10, both upregulated in the female antenna showing an increase > 200%. In contrast, the ORs upregulated in the male antenna were not grouped into clusters (Fig. 4).

##### Ionotropic receptors

Phylogenetic analysis showed a wide distribution of *D. longicaudata* IRs in clades/clusters represented by *D. alloeum* and *Apis mellifera*. Based on the phylogenetic analysis and direct orthology to *D. alloeum* sequences, possible clade expansions for IRs 64a, 64a5, 126 and 138 were observed in *D. longicaudata* (Figure S2).

The total number of transcripts per group (female or male) expressed in the antenna of *D. longicaudata*, and the percent of those transcripts per expression range (low, medium, high) is provided in Fig. 1. Among the three ionotropic co-receptors (IRcos), namely IRs 8a, 25a and 76b, the first showed the highest expression in the antenna of both sexes (Table S1: Sheets D and E). No differential expressed IRs were found between female and male antennae (Table S4: Sheet A).

##### Gustatory receptors

The GR phylogenetic analysis is presented in Figure S2, and no clade expansions were found for this gene family. The expression of most GRs in the antenna of both sexes was low (Fig. 1), with only GR UNCH-2 showing medium expression (Table S1: Sheets D and E). No differentially expressed GRs were found between sexes (Table S4: Sheet A).

#### Odorant-molecule carriers

##### Odorant-binding proteins

This chemosensory family was the most expressed group (on average) in the *D. longicaudata* antenna; six sequences in females and five in males of a total of 12 OBPs showed a high transcript expression level and only three OBPs in the female and two in the male antenna showed low expression (Fig. 1; Table S1: Sheets D and E). The OBP 1 showed the highest expression in the antenna of both sexes, and the OBP UNCH-1 was upregulated in the male antenna (Fig. 2; Table S4: Sheet A), showing a 24-fold increase in males with respect to females.

Based on the amino acid sequence, the number of conserved cysteines observed in all identified OBP sequences positioned them in the classic OBP subtype group (Data file S1). The phylogenetic analysis suggested clade expansions for OBPs 7 and 11 (Figure S2).

##### Chemosensory proteins

This gene family was the second most expressed group in the *D. longicaudata* antenna (Fig. 1; Table S1: Sheets D and E). However, there was only one sequence (CSP 4) with medium expression and another (CSP 5) showing high transcript expression level in the antenna of both sexes (Fig. 1; Table S1: Sheets D and E). Both sequences were upregulated in the male antenna (Fig. 3; Table S4: Sheet A). All sequences had the four conserved cysteines, a common feature of this gene family (Data file S1).

##### Niemann-pick disease type C2 like proteins

These odor-molecule carriers showed a transcript expression level from medium to high in the *D. longicaudata* antennae and were particularly highly expressed in males (Fig. 1; Table S1: Sheets D and E). However, no differentially expressed NPC2-like proteins were found between the female and male antennae (Table S4: Sheet A). In addition, no clade expansions were detected and direct orthology was found for all *D. longicaudata* CSPs and NPC2-like proteins to sequences of *D. alloeum* (Figure S2).

#### Sensory-related ion channels

##### Pickpocket receptors

This gene family showed the lowest expression of all sensory gene families analyzed here. All PPKs showed low expression (Fig. 1; Table S1: Sheets D and E), and no differentially expressed PPKs were identified (Table S4: Sheet A).

##### Transient receptor potential channels

Five TRP genes in females and three in males (out of a total of 16 TRPs annotated) had a medium expression level (Fig. 1; Table S1: Sheets D and E). The TPR_LK (belonging to TRPC subfamily) showed the highest expression in the female antenna (Table S1: Sheets D and E). The same receptor was upregulated between the female and male antenna (Fig. 3; Table S4: Sheet A), showing a ~ 5000-fold increase in females with respect to males (Table S1: Sheets D and E), and almost not expressed in males.

The phylogenetic analysis showed no clade expansion for PPKs and a possible clade expansion for the TRPs UNCH-1, 5, 6, 8, 9 and 11 (Figure S2).

#### Other sensory-related gene families

##### Ammonium transporter receptors

Two AMTs were identified in the antenna of *D. longicaudata* and one of them showed a medium expression level, namely AMT Rh-typeA (Fig. 1; Table S1: Sheets D and E). Neither of these two AMTs showed differential expression between male and female antennae (Table S4: Sheet A).

##### CD36 and sensory neuron protein receptor gene family

Seven members of this gene family (out of a total of 12 receptors identified), showed a medium expression level in the antenna of both sexes (Fig. 1). Only SNMP 1.1 showed high expression level in the antenna of both sexes and SR-B1 UNCH-1 in the female antenna (Table S1: Sheets D and E). No member of this gene family showed differential expression between the female and male antennae (Table S4: Sheet A).

The phylogenetic analysis showed direct orthology for all CD36/SNMPs and AMTs identified here to ortholog sequences of *D. alloeum*, and no clade expansions were found for both gene families (Figure S2).

### Gene ontology (GO) enrichment analysis

**Fig. 6:**
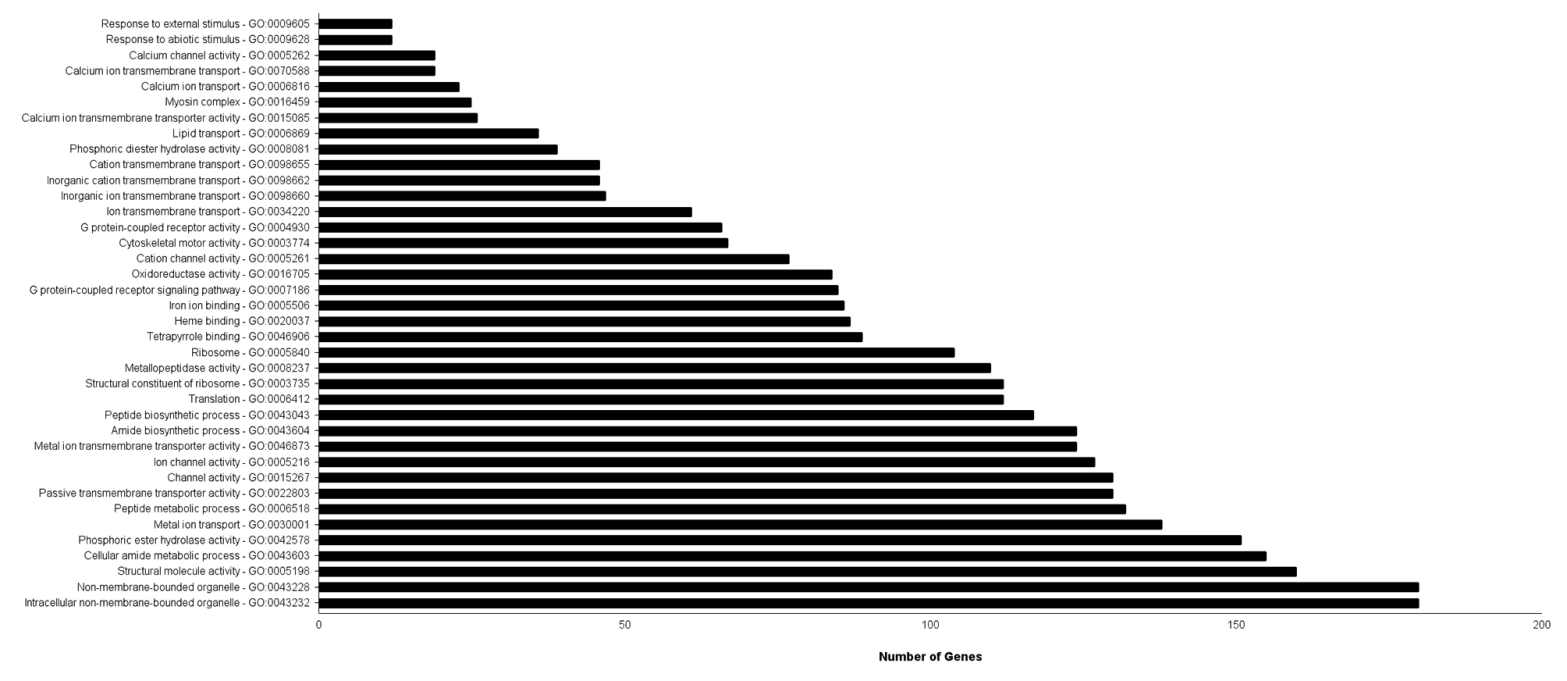
Gene Ontology (GO) enrichment analysis. The GO-terms enriched in the female antennae (vs. male antennae) are presented here. X-axis and y-axis represent the enriched GO-terms (corrected *P*-value < 0.05) and the number of genes per GO-term, respectively. A total of 13,342 genes were included in the analysis, and detailed information on the analysis is given in Table S5

Regarding the genes associated with GO-terms enriched in the female antennae (vs. male antennae) directly or indirectly associated with sensory activity, 130 genes were classified into channel activity (GO:0015267) and 127 into ion channel activity (GO:0005216). In addition, 61 and 47 genes, included in the enriched terms GO:0034220 and GO:0098660, respectively, were associated with ion trans-membrane transport. Further, 138 and 124 were specific to metal ions, GO:0030001 and GO:0046873, respectively; 77, 46 and 46 genes specific to cations, GO:0098655, GO:0005261, and GO:0098662, respectively, and 26, 23, 19 and 19 genes specific to calcium transport (GO:0015085, GO:0006816, GO:0005262 and GO:0070588, respectively) (Fig. 6; Table S5).

Among other enriched terms potentially associated with stimuli sensing, 85 genes were grouped under the G protein-coupled receptor signaling pathway, and 66 in G protein-coupled receptor activity, including GO:0007186 and GO:0004930, respectively. In addition, 12 genes were associated with response to external stimulus and another 12 genes with response to abiotic stimulus, GO:0009605 and GO:0009628, respectively (Fig. 6; Table S5). Several terms associated with CYP activity were identified: GO: 0046906 associated with tetrapyrrole binding (including 89 genes), GO:0020037 associated with heme binding (including 87 genes) and GO; 0005506 associated with iron ion binding with 86 genes (Fig. 6; Table S5).

## Discussion

The present study contributed to the characterization of the sensory-related genes of the parasitoid wasp *D. longicaudata*, and suggested candidate genes associated with the female host-seeking behavior in this species. Ten sensory-related gene families expressed in the antenna of *D. longicaudata* were annotated. The number of sequences identified was like that observed for other hymenopteran species, Table 1 [11, 19, 20, 25, 48–50]. The quality of the assembled transcriptome suggests that most of the sensory genes expressed in the antenna of *D. longicaudata* during host finding were identified. Among these, complete sequences of most of the characterized transcripts were obtained, however 6% of these (18 ORs, 3 GRs and 1 OBP) were not fully reconstructed. Further analyses are needed, including the *D. longicaudata* whole genome sequencing, to clarify this point. For instance, our results do not match the number of sensory-related genes previously identified in a transcriptome of pooled tissues of *D. longicaudata* [47]. In the mentioned study, the authors reported 43 OBPs, 69 CSPs, 60 GRs, 689 ORs 26 IRs, and 14 SNMP [47]. These are much higher numbers compared with other hymenopteran species, see Table 1 [11, 19, 20, 25, 48–50]. The differences in the number of genes between the two studies may be related to the fact that CDS sequences with similarity > 95% were grouped into clusters to remove redundancy in our antennal transcriptome. However, in the mentioned work the number of duplicated BUSCOs was 90% vs. 10% in our transcriptome. Below we will discuss the results associated with the sensory-related genes differentially expressed (DE) between the female and male antenna of *D. longicaudata*. However, the non-sensory genes DE between the antenna of both sexes will be addressed in more detail in a future work.

**Table 1:** Transcribed gene sequences associated with chemoreception in *D. longicaudata* and other taxonomically related species

Species	OR	IR	GR	CSP	OBP	AMT	PPK	TRP	CD36/ SNMP	NPC2- like	Citation
*Diachasmimorpha longicaudata*	222	44	25	10	15	2	8	16	12	4	Wulff et al. 2021; This study
*Diachasma alloeum*	187	51	39	9	15	2	7	12	11	4	Tvedte et al. 2019
*Apis mellifera*	163	10	10	6	21	2	8	12	12	4	Foret et al. 2007
*Nasonia vitripennis*	225	99	47	9	90	2	3	14	5	5	Robertson et al. 2010
*Microplitis demolitor*	218		85							2	Zhou et al. 2015
*Microplitis mediator*		17		3	20					3	Wang et al. 2016; Zheng et al. 2018

Ninety-three of a total of 97 sensory-related transcripts that were differentially expressed between both sexes were OR transcripts, and 87 of those 93 were upregulated in the female antenna and five downregulated. Our results agree with studies on other parasitoid wasps such as *Cotesia vestalis* Haliday (Hymenoptera: Braconidae), *M. mediator* and *Chouioia cunea* Yang (Hymenoptera: Eulophidae), where most of the differentially expressed chemoreceptors between the female and male antennae were ORs [12, 14, 51]. The upregulated ORs in the *D. longicaudata* female antenna were distributed into 28 clades, six of which included three or more upregulated sequences. Our results, in accordance with other studies in parasitic wasps [12, 14, 52], seem to highlight three relevant aspects regarding the OR expression in the antenna of parasitoid wasps: (1) the number of ORs upregulated in the female antenna is always much higher than those upregulated in the male antenna; (2) most of upregulated ORs in the female antenna were clustered by phylogenetic analysis, whereas those few upregulated in the male antenna tend to be scattered, which could suggest that the latter are associated with unrelated functions; (3) most of the upregulated receptors in the female antenna tend to be associated with clade expansions. In summary, OR clade expansions in conjunction with differential gene expression could be a strategy present in wasps [53] and other hymenopteran species [54] associated with specialized sensory demands within particular behaviors, e.g. host-seeking behavior.

Aside from the ORs, the TRPL gene was the only other upregulated transcript in the female antenna. TRPL was associated with light responses and thermal sensing in *D. melanogaster* [55]. In the parasitoid wasp *Cotesia congregata* [56], it was observed that high temperatures affect the development of the parasitoid within the host and also the development of the host itself [57, 58]. Consequently, the high expression of TRPL in the female antenna could be related to the detection of suitable microhabitats for oviposition in terms of appropriate temperature.

Odorant-molecule carriers (CSPs, OBPs) were the most highly expressed transcripts in the antenna of *D. longicaudata*. Both gene families are normally highly expressed in the insect antenna assisting and modulating the chemodetection process [7]. We found two CSPs (CSP 4 and CSP 5) and one OBP (OBP UNCH-1) upregulated in the male antenna. These genes could have a role in female pheromone detection and/or act in association with those five ORs that were also upregulated in the male antenna. *D. longicaudata* and other parasitoid wasps, such as *Aphidius colemani* Esenbeck (Hymenoptera: Braconidae), are monandrous species, which means a single mate in a lifetime [59, 60] However, it was observed that males actively court both mated and virgin females, indicating that the production of female-borne sex attractants does not cease or strongly diminish immediately after mating [59–61]. Many CSPs, OBPs and ORs have been previously associated with sex pheromone detection in other insect species [62, 63]. In addition, we can expect that those receptors associated with food detection will not be differentially expressed between sexes in *D. longicaudata* (as we will discuss below), consequently, the receptors upregulated in the male antenna could be associated with female pheromones detection. Future assays that analyze the gene expression of CSPs, OBPs and ORs in *D. longicaudata* males before and after mating may shed light on the expression and function of these genes.

According to our phylogenetic analysis, no functional information is available in those species close to *D. longicaudata* carrying the orthologs of the sensory-related genes (67 ORs, 1 TRP, 2 CSPs and 1 OBP) we found differentially expressed between the female and male antenna. We also avoided establishing hypotheses based on functional information of those receptors from species not included in the phylogenetic analysis to define a subgroup of *D. longicaudata* receptors to move forward with future functional assays. Those species could have completely different feeding, mating, or host-seeking behaviors. Through our methodological approach, using mature/mated adults without previous experience in presence of the host (naïve females), we expected that those sensory-related receptors associated with the innate detection of the host and host habitat will be upregulated in females, but not those associated with mating or feeding behaviors. For instance, the high number of OR transcripts upregulated in the female antenna (87), suggest that at least part of those upregulated ORs could have a central role in females associated behaviors such as host-seeking for oviposition. We cannot confirm this hypothesis based on our phylogenetic analysis, however, if we focus on the biology of *D. longicaudata* and other parasitoid wasps, we can define a subgroup of ORs to proceed with functional assays to test the hypothesis that the majority of the upregulated ORs in the female antenna of *D. longicaudata* are associated with host-seeking behavior.

Mating status seems to play an important role in modulating the host-seeking behavior in parasitoid wasps. It was observed for some parasitoid species that mated females were more attracted to the host compared to the virgin ones [64]. As we mentioned above and expected for a solitary parasitic wasp, *D. longicaudata* is a monandrous species [59]. Nevertheless, under mass-rearing conditions remating was observed for solitary parasitoids including *D. longicaudata* [61]. This behavior may not happen in the field, but it was neither confirmed nor disproven. For instance, Benelli [60] observed for *A. colemani* (another monandrous wasp) that mated females refuse to copulate more than once. Consequently, we could suggest that a change in the expression of some sensory-related genes should occur before and/or during the mating process and remain relatively unchanged until the female gains experience in the presence of the host/host habitat. We are planning to run further analysis, using virgin, mated/naïve and females in the presence of the host to test this hypothesis.

Host-seeking behavior in female parasitoids is a complex process that involves different chemical and physical signals from the environment and the host [65]. As expected for a generalist parasitoid [66], the response of *D. longicaudata* naïve females to chemical cues becomes more complex as they accumulate experience with different hosts and host habitats [45]. In line with those observations, Ballesteros [67] found in *Aphidius ervi* Haliday (Hymenoptera: Braconidae), that some chemosensory genes were upregulated in parallel to the female experience in the presence of the host and host-habitat cues. Upregulation of sensory-related genes of *D. longicaudata* could be also driven in parallel to the gain of experience by a female wasp. However, we focused our study on mated/naïve females to address only those sensory-related genes associated with innate detection of the host and host habitat. The ultimate goal of this study was to identify sensory-related genes associated with the detection of the host during the first days that the naïve females are released into the field for pest control because the survival of the parasitoids decreases as the days progress.

The ORs that we found upregulated in the female antenna of *D. longicaudata* could be associated with the search for specific nutrients necessary to complete oogenesis. *D. longicaudata* is a synovigenic hymenopteran parasitoid [68], and females of synovigenic species emerge with only a fraction of their total egg complement as mature eggs, in contrast to proovigenic wasps which complete oogenesis before adult female emergence [69]. Synovigenic parasitoids are generally idiobionts and attack primarily host eggs and pupae, however, *D. longicaudata* is a koinobiontic endoparasitoid, and lays eggs in late larval stages. Koinobiont wasps have higher longevity than idiobiontic species [69], and to sustain oogenesis, parasitoid females require additional nutrients [69, 70]. However, *D. longicaudata* females and males only feed on nectar flower [70] or honey under mass-rearing conditions [71]. To complete oogenesis, some parasitoid species such as *D. longicaudata*, produce hydropic eggs, which complete embryonic development in the host following oviposition [68]. In addition, many parasitoids feed on flowers of plants not infested by the parasitoid host [70]. It was observed that the size and configuration of the flowers determined which flowers were used as a food source and the accessibility to nectar was more important than the chemicals released by them [70, 72]. In this sense, Desurmont [73], found in the parasitoid *Cotesia glomerata* Linnaeus (Hymenoptera: Braconidae), that the floral odors can act as background contaminants reducing the attraction of the parasitoid females to the plants infested by the host. This evidence suggests that the upregulated ORs in the female antenna would not be associated with the feeding process.

Regarding the main chemical signals that parasitoid wasps use to find the host, they are mainly guided by natural plant semiochemicals, host kairomones and microbial volatiles associated to decaying vegetal tissues [74]. *D. longicaudata* female seems to have an innate host habitat preference [45] even when the fruit was not infested, but showed signs of fruit decay [3, 75]. Further, Carrasco [76] found the same compounds shared by infested and non-infested fruits, but a higher dose in the former, and suggested that the compound concentration could also be an important element in guiding the host-seeking behavior of *D. longicaudata* females. Consequently, we can suggest that the ligands of the receptors that we found upregulated in the female antenna are mainly volatile organic compounds (VOCs) released by decomposing fruits and/or those found in different fruit conditions but at higher concentrations. Our futures studies will focus on specific chemicals released by rotten fruits and/or fungi and bacteria associated to rotten fruit, such as 1-Butanol [77]. Those studies will include a gene silencing approach, as a strategy to identify possible ligands for the top upregulated ORs in the female antenna of *D. longicaudata*. In terms of candidate genes, we plan to prioritize those ORs included in clades 1 and 2 (see Results section), which include many ORs upregulated in the female antenna and some of them showed a > 200% increase with respect to the male antenna.

We did not find differentially expressed genes for the other sensory-related families analyzed in the present study. Following, we will mention only a few receptors among the most highly expressed genes in the antenna of both sexes, which could be addressed in future works. Regarding ionotropic receptors, IRs included in the clusters 64a and 75u were the most highly expressed within this group. IR 64a receptors are grouped as antennal IRs [78] and were associated with CO_2_ and acid detection in *D. melanogaster* [79], and IRs 75u-like showed high expression in the antennae of several hymenopteran species [12, 13, 48], however the role of these genes has not been determined by functional tests in parasitoid species. Among CD36/SNMPs, SNMP 1.1 was one of the most highly expressed SNMP. This gene is an ortholog of the *A. mellifera* SNMP 1.1, was detected in the antenna of many species and has been extensively studied in insects in association with pheromone detection [41, 80–82]. Regarding AMTs, Amt Rh-typeA was the most highly expressed ammonium transporter. This receptor was detected highly expressed in the olfactory neurons of *D. melanogaster* [83] and was suggested as a non-canonical odorant receptor for ammonia [28]. Ammonia is a decomposition derivative of protein and together with polyamines such as putrescine (abundant in rotten fruits), represents two of the primary attractants for tephritid fruit flies, which are the *D. longicaudata* hosts [84]. Consequently, this receptor could be associated with the recognition of ammonia and other nitrogen derivates involved in host habitat detection. GRs together with PPKs showed the lowest expression in the *D. longicaudata* antennae of both sexes. The second most highly expressed GR (GR 3), showed direct orthology to the *A. mellifera* GR 1, which is associated with fructose sensation [85]. This receptor could have a role in food detection since individuals of both sexes feed on flower nectar [86]. Among PPKs, PPK 23 and PPK 28 were among the most highly expressed PPKs in *D. longicaudata* antenna. There is no functional information available for the orthologs of these genes in species closely related to *D. longicaudata*. However, in *D. melanogaster*, PPK 23 and 28 were found to be expressed in gustatory signal neurons [87] and silencing of these receptors modified the male response to female pheromones [62]. Consequently, these PPK receptors could have a role in mating behavior.

## Conclusions

Our findings provide insight into the molecular mechanisms associated with sensory-related genes at the antennal level in sexually mature and mated females of *D. longicaudata*. Our results suggest that the odorant receptor gene family has a central role in the detection of chemical cues of suitable hosts for oviposition or their habitats. This study constitutes a starting point to characterize the repertoire of sensory-related genes associated with the host-seeking behavior for oviposition in the parasitoid wasp *D. longicaudata.* Further, based on the comparison of gene expression patterns, we were able to postulate ORs potentially associated to mating behavior in males. We plan to run future functional assays to test the hypotheses discussed in the present study.

## Methods

### Biological material

The parasitoid wasps and fruit flies used in the study were reared at the Laboratorio de Insectos de Importancia Agronómica, Instituto de Genética Ewald A. Favret gv-IABIMO, Instituto Nacional de Tecnología Industrial (INTA) and Consejo Nacional de Investigaciones Científicas y Técnicas (CONICET). *D. longicaudata* individuals were obtained from a colony named IGEAF_*D.longicaudata*-Af, reared on *Anastrepha fraterculus* Wiedemann for 30 generations [25]. This colony was established from *D. longicaudata* wasps reared on *Ceratitis capitata* Wiedemann for ~ 200 generations, named IGEAF_*D.longicaudata*-Ca_colony, which was established with parasitoids provided by Fundación Miguel Lillo-CIRPON [71]. Insects were maintained under controlled rearing conditions at 25 ± 2°C, 65 ± 5% relative humidity and a 12:12 (light/dark) photoperiod, following protocols described by [88]. Biosecurity considerations complied with CONICET resolution 1619/2008 and with the Worldwide Health Organization (WHO) Biosecurity Handbook (ISBN 92 4354 6503).

### Behavioral trial for the selection of *D. longicaudata* host-seeking females

Sexually mature and mated females (7-d-old) with no experience in oviposition and sexually mature and mated males (7 to 9-d-old) were selected as follows. Females were collected by conducting a behavioral trial in which those females attracted to the host (*A. fraterculus* L3 larvae) were chosen as host-seeking females. To perform the trial, a total of 1000 larvae were offered to *D. longicaudata* wasps (7-d-old females and males mixed) that were placed inside a cage (cage 1; dimensions 40 × 40 × 40 cm). Larvae were placed in three Oviposition Units (OUs), each consisting of a Petri dish, placed upside down on a second cage (cage 2; same dimensions as cage 1), and cage 2, above and connected with cage 1 through a small opening of ~ 10 × 10 cm, to let the females move from cage 1 to cage 2. A visual barrier was placed covering the OU to block the use of visual cues from the parasitoid females. Only the females that reached the OU during the first 15 min were selected for the molecular experiment, while 7/9-d-old males (not attracted by the host) were randomly collected from cage 1.

### Tissue dissection and total RNA isolation

Total RNA was extracted from an antenna homogenate of *D. longicaudata* for each group. The groups were as follows: (1) antenna of female and (2) antenna of male. Each group included *n* = 5 replicates, and each replicate included 60 pooled antennae, either from female or male individuals. Female and male antennae were cut using dissection vannas scissors (WPI, Worcester, MA, USA), immediately after being placed in 600 µl of cold Trizol^®^ reagent kept on ice (Invitrogen, Carlsbad, CA, USA) and stored at -80°C until use. Total RNA was extracted according to the manufacturer’s specifications. The integrity of the extracted RNA was tested using 1% agarose gel electrophoresis and quantified using a Nanodrop-1000 spectrophotometer (Thermo-Fisher Scientific, Waltham, MA, USA).

### RNA-seq processing and data analysis

Library construction and sequencing services (paired-end 150 bp using an Illumina NovaSeq 4000) were carried out by Novogene Corporation, Inc. (Sacramento, CA, USA). Briefly, for library construction, RNA samples were enriched using oligo(dT) beads (Illumina, San Diego, CA, USA). Then, the mRNA was fragmented randomly using a fragmentation buffer (Illumina), followed by the first strand cDNA synthesis using mRNA as a template, random hexamer primers, a custom buffer (Illumina), dNTPs (Invitrogen) and DNA polymerase I (Invitrogen). The second strand was synthesized after a RNAse H treatment (Invitrogen). Finally, after the sequence terminal repair, sequencing adaptors were ligated (Illumina). The double-stranded cDNA library was completed through size selection and PCR enrichment.

FastQC v0.11.5 software tool (http://www.bioinformatics.babraham.ac.uk/projects/fastqc/) was used to assess read quality and detect Illumina adaptors. Subsequently, Trimmomatic v0.36 [89] was used to trim off low-quality bases at the 5′ and 3´ends using the parameters as follows: trailing: 5; leading: 5; and sliding-window 4:15. Reads shorter than 50 bp and adaptors were removed using the parameters ILLUMINACLIP: TruSeq3-PE-2.fa:2:30:10.

### Transcriptome assembly and prediction of coding sequences

The quality-filtered and trimmed reads from the 10 libraries were used to assemble a *de novo* assembly by means of Trinity v2.10.0 [90]. The *TrinityStats.pl* script was then applied to generate the basic statistics of the assembly. A non-redundant coding sequence (CDS) database of the assembled transcripts was accomplished following the strategy used by [91]. Initially, Open Reading Frames (ORFs) of at least 100 amino acid length were predicted using the *TransDecoder.LongOrfs* script from the TransDecoder v5.5.0 (http://transdecoder.github.io). Next, BLASTp v2.9.0 + and HMMscan v3.2 searches were conducted on the predicted ORFs using the complete UniProtKB/Swiss-Prot and Pfam-A databases as queries. The results of these searches were used in the *TransDecoder.Predict* script to obtain the predicted CDS. Then, CDS with similarity > 95% were grouped into clusters, keeping only one representative sequence per cluster using the *cd-hit-est* script of CD-HIT v.4.8.1. The resulting non-redundant CDS database was used to conduct the differential expression analysis, and the protein sequences obtained from its translation were used to identify the sensory gene repertoire of *D. longicaudata.* The completeness of the non-redundant translated CDS database was analyzed through BUSCO v4.1.4, in protein mode, against the hymenoptera_odb10 data set.

### Sensory-related transcript identification, sequence manual curation and phylogenetic analyses

Protein sequences from *D. melanogaster*, *A. mellifera*, *Nasonia vitripennis* Walker (Hymenoptera: Pteromalidae), *Microplitis demolitor* Wilkinson (Hymenoptera: Braconidae), *Microplitis meditator* Haliday (Hymenoptera: Braconidae), *D. alloeum*, and *F. arisanus* were used in local BLASTp v2.9.0 + searches against non-redundant translated CDS database of *D. longicaudata* to identify sensory-related transcripts. After sequence identification, NCBI-tBLASTn and BLASTp searches against NCBI nucleotide collection and UniProtKB/Swiss-Prot databases, respectively, were conducted for each sensory-related gene family. The Expect threshold was set at 0.05 and only the top 10 hits were recorded for both searches.

The myeloid differentiation factor 2 (MD-2)-related lipid-recognition proteins have not been extensively studied in insects and have been classified into different sub-groups such as Niemann–Pick disease type C2 (NPC2), ecdysteroid regulated 16 kDa (ESR16) and myeloid differentiation factor 2 (MD-2) proteins [7, 17, 20, 25, 92, 93]. NPC2s are the most widely studied members of this family of proteins in insects [17, 20, 92, 93]. For ease of reading, here we grouped and designated NPC2s, ESR16s and MD-2s as NPC2-like proteins. Further, the CSPs, OBPs and NPC2-like proteins previously reported for *D. longicaudata* [25] were used to assist the manual curation of the identified sequences.

*D. longicaudata* sensory-related protein sequences, as well as those protein sequences from the genome of *A. mellifera* and transcriptome of *D. alloeum*, were used for phylogenetic analysis. The amino acid sequences from each family were aligned using MAFFT [94] with the G-INS-1 strategy and the following settings: unaligned level = 0.1; offset value = 0.12; maxiterate = 1000 and the option *leave gappy regions*. Afterward, the alignment was trimmed using trimAl v1.2 [95] by default, except for the gap threshold set at = 0.3. The resulting alignments were used to create phylogenetic trees on IQ-tree v1.6.12 (http://www.iqtree.org/). The branch support was estimated using the approximate Likelihood Ratio Test based on the Shimodaira-Hasegawa aLRT-SH [96]. ModelFinder [97] was used to establish the best-fit amino acid substitution models and chosen according to the Bayesian Information Criterion. These models were JTT + F + R9 for ORs, JTT + F + R4 for GRs, JTT + F + R5 for IRs, LG + I + G4 for CSPs, LG + R3 for OBPs, WAG + F + R3 for PPKs, VT + R4 for TRPs, LG + R3 for CD36/SNMPs, cpREV + G4 for AmT and WAG + G4 for NPC2. The phylogenetic trees were visualized and edited with iTOL v6 (https://itol.embl.de/). Gene candidates were annotated according to their phylogenetic relation to sequences of *D. alloeum*.

Only complete and partial sequences at least 70% complete in comparison with orthologous sequences from other insect species mentioned above, were used in the phylogenetic analysis. Partial and assembled sequences (from more than one transcript) were defined using manual curation based on ClustalW alignments between *D. longicaudata* protein sequences and those orthologous sequences from the insect species mentioned above.

Transcripts per kilobase per million (TPM) values were estimated and used in the gplot package v.3.1.1 to create heatmaps and visualize transcript abundance for the target gene families. Transcript expression levels were defined following the EMBL-EBI guidelines, where a TPM value < 0.5 is considered to have no expression at all, TPMs ≥ 0.5 to ≤ 10 is equal to low expression, TPMs ≥ 11 to ≤ 1000 is medium expression, and TPMs > 1000 is equivalent to high expression (https://www.ebi.ac.uk/gxa/home). However, the TPM per transcript and family was plotted as Log_10_ (TPM + 1) to obtain a range of TPM gene transcripts that can be accommodated on the same graph.

The sensory protein sequences identified were annotated and named according to direct orthology to a single sequence of the species used for the phylogenetic analyses (see above). Those sequences for which no direct orthology to a single sequence was achieved were annotated as uncharacterized receptors, *“*DlonOR_UNCH”, and numbered following a sequential order.

### Transcript quantification and differential expression analysis

The *align_and_estimate_abundance.pl* Trinity script and Salmon v1.4.0 [98] were used to map the trimmed and cleaned reads from each library to the non-redundant CDS database. Next, the count matrix per transcript for each sample was generated by means of the *abundance_estimates_to_matrix.pl* Trinity script. Highly variable and/or low-expression transcripts were deleted from this matrix using HTSFilter v.1.28 [99] in RStudio. The resulting matrix was the input of DESeq2 v.1.28.0 [100] to carry out the differential expression analysis in RStudio through the *apeglm* estimation from the *lfcShrink* function that uses an adaptive Bayesian shrinkage estimator to generate more accurate Log_2_ Fold-Change (Log_2_FC) values. An s-value < 0.05 as a significance level and an Log_2_FC > 1, equal to an expression change of 100%, were used to identify differentially expressed transcripts between female and male antennae. An expression change of 100% was also determined as significantly differentially expressed gene in a similar study performed in a close species to *D. longicaudata* [67].

Sequences obtained from the DGE analysis (in which sensory genes were not included, since these genes were identified as mentioned above) were subsequently submitted to BLASTp searches against UniProtKB/Swiss-Prot and a customized protein database formed by sequences (downloaded from: https://ftp.ncbi.nlm.nih.gov/genomes/refseq/invertebrate/) from *A. mellifera*, *D. alloeum*, *D. melanogaster, F. arisanus*, *M. demolitor* and *N. vitripennis*. In addition, HMMscan searches were performed using the Pfam-A database. The results of these searches were combined using Trinotate v3.2.1 (https://trinotate.github.io).

### Gene ontology enrichment analysis

The Gene Ontology (GO) terms from the sequences of the non-redundant translated CDS database were locally obtained using InterProScan 5 (https://github.com/ebi-pf-team/interproscan). The GO enrichment analysis was performed using ermineR by means of the Gene Score Resampling (GSR) method that uses the absolute logFC values for all genes to produce a score rank. This rank is analyzed as a continuous variable, and it examines GO-terms that are enriched. More information about this method is available at https://erminej.msl.ubc.ca/help/tutorials/running-an-analysis-resampling. Those GO-terms with a corrected *P*-value < 0.05 were considered enriched in our antennal transcriptome.

### Electronic supplementary material

Below is the link to the electronic supplementary material.

Supplementary Material 1

Supplementary Material 2

Supplementary Material 3

Supplementary Material 4

Supplementary Material 5

Supplementary Material 6

Supplementary Material 7

Supplementary Material 8

Supplementary Material 9

## Data Availability

All data generated or analyzed during this study are included in this published article (and its supplementary information files). The raw sequence dataset is available at the National Center for Biotechnology Information (NCBI) under the SRA Bioproject number PRJNA853733.

## Abbreviations

aa: amino acids
AMT: Ammonium Transporter
BLAST: Basic Local Alignment Search Tool
BUSCO: Benchmarking Universal Single-Copy Orthologs
CDS: Coding Sequence
CONICET: Consejo Nacional de Investigaciones Científicas y Técnicas
CSP: Chemosensory Protein
DGE: Differential Gene Expression
FC: Fold-Change
Gb: Gigabyte
GC: Guanine-Cytosine
GO: Gene Ontology
GSR: Gene Score Resampling
GR: Gustatory Receptor
INTA: Instituto Nacional de Tecnología Industrial
IR: Ionotropic receptor
L3: 3rd-instar Larva
NCBI: National Center for Biotechnology Information
NPC2-like: Niemann-Pick type C2 like protein
OBP: Odorant-Binding Protein
ODE: Odorant Degrading Enzyme
ORF: Open Reading Frame
OR: Odorant Receptor
OSNs: Olfactory Sensory Neurons
OU: Oviposition Unit
PCA: Principal Component Analysis
RNA: Ribonucleic Acid
SRA: Sequence Read Archive
PPK: *Pickpocket* Receptor
CD36/SNMP: Cluster of Differentiation 36 / Sensory Neuron Membrane Protein
TN: TRINITY
TPM: Transcripts Per Million
TRP: Transient Receptor Potential channel
WHO: Worldwide Health Organization
