# Supplementary material for: Identification of candidate genes associated with host-seeking behavior in the parasitoid wasp Diachasmimorpha longicaudata
Source: BMC Genomics. 2024 Feb 6;25:147. doi: 10.1186/s12864-024-10034-6 (PMC10848486; doi:10.1186/s12864-024-10034-6)

Additional File 2: Supplementary Figure S1. BUSCO analysis results. A total of 33,746 CDS were analyzed. Abbreviations: CDS: coding sequences; S: single copy; D: duplicated; F: fragmented; M: missed; n: total number of genes.

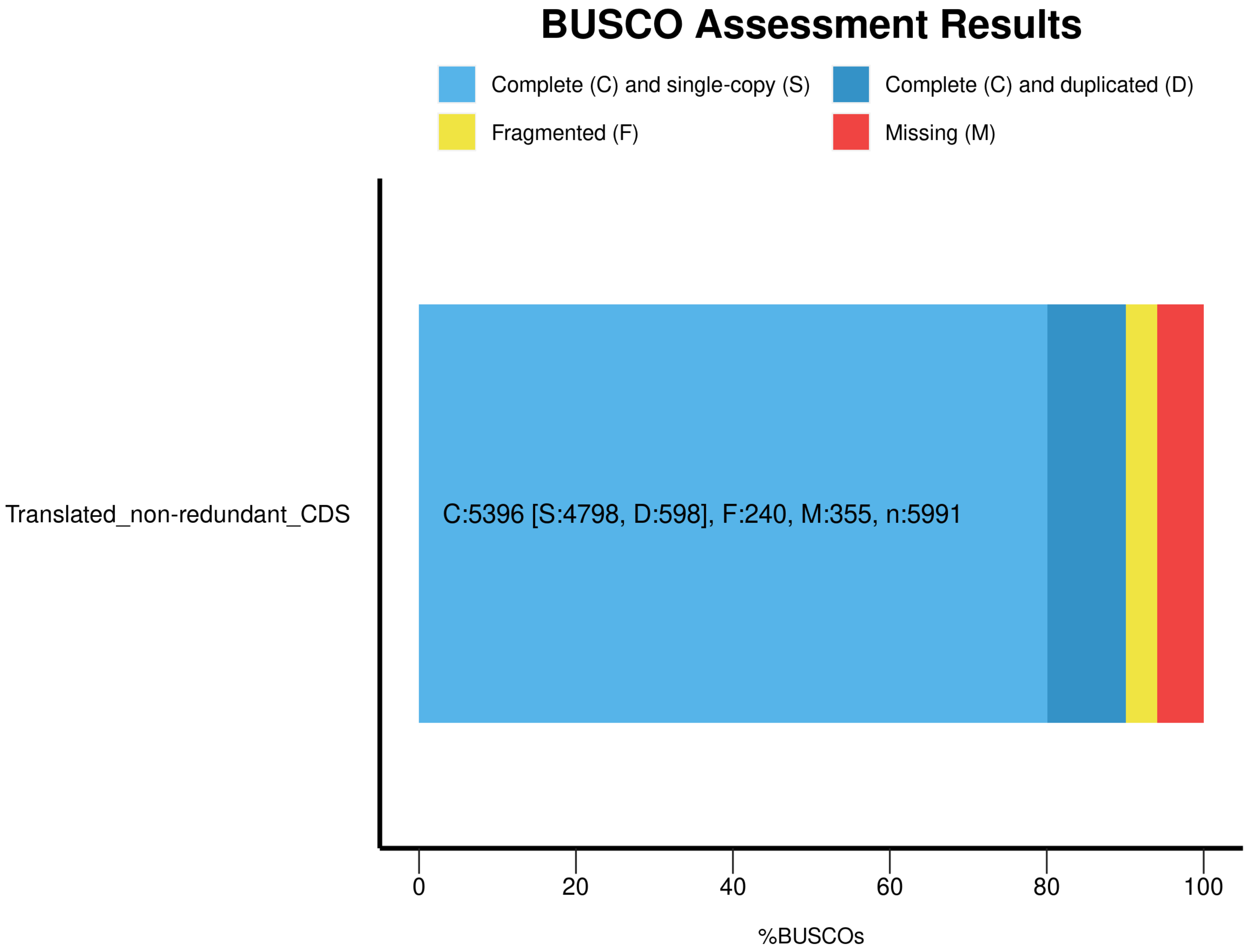

Supplement: Supplementary file 2 — Supplementary Material 2 [file 12864_2024_10034_MOESM2_ESM.pdf]
