# Supplementary material for: Identification of candidate genes associated with host-seeking behavior in the parasitoid wasp Diachasmimorpha longicaudata
Source: BMC Genomics. 2024 Feb 6;25:147. doi: 10.1186/s12864-024-10034-6 (PMC10848486; doi:10.1186/s12864-024-10034-6)
Supplement: Supplementary file 5 — Supplementary Material 5 [file 12864_2024_10034_MOESM5_ESM.pdf]

Additional File 5: Supplementary Figure S2. Phylogenetic analysis by Maximum Likelihood estimation of sensory-related gene families. Those receptors up- or downregulated between the female and male antenna were differentiated with a red and blue tag, respectively. Color code: *D. longicaudata* (Dlon, red), *D. alloeum* (Dall, blue), *A. mellifera* (Amel, yellow), and *T. castaneum* as a outgroup (Tcas, black). (**A**) ammonium transporters (AMTs); (**B**) chemosensory proteins (CSPs); (**C**) gustatory receptors (GRs); (**D**) ionotropic receptors (IRs); (**E**) Neimann-Pick type C2 like proteins (NPC2-like); (**F**) odorant-binding proteins (OBPs); (**G**) pickpocket receptors (PPKs); (**H**) CD36-sensory neuron membrane proteins (CD36/SNMPs); and (**I**) transient receptor potential channels (TRPs). Abbreviations: LK: like, UNCH: uncharacterized.

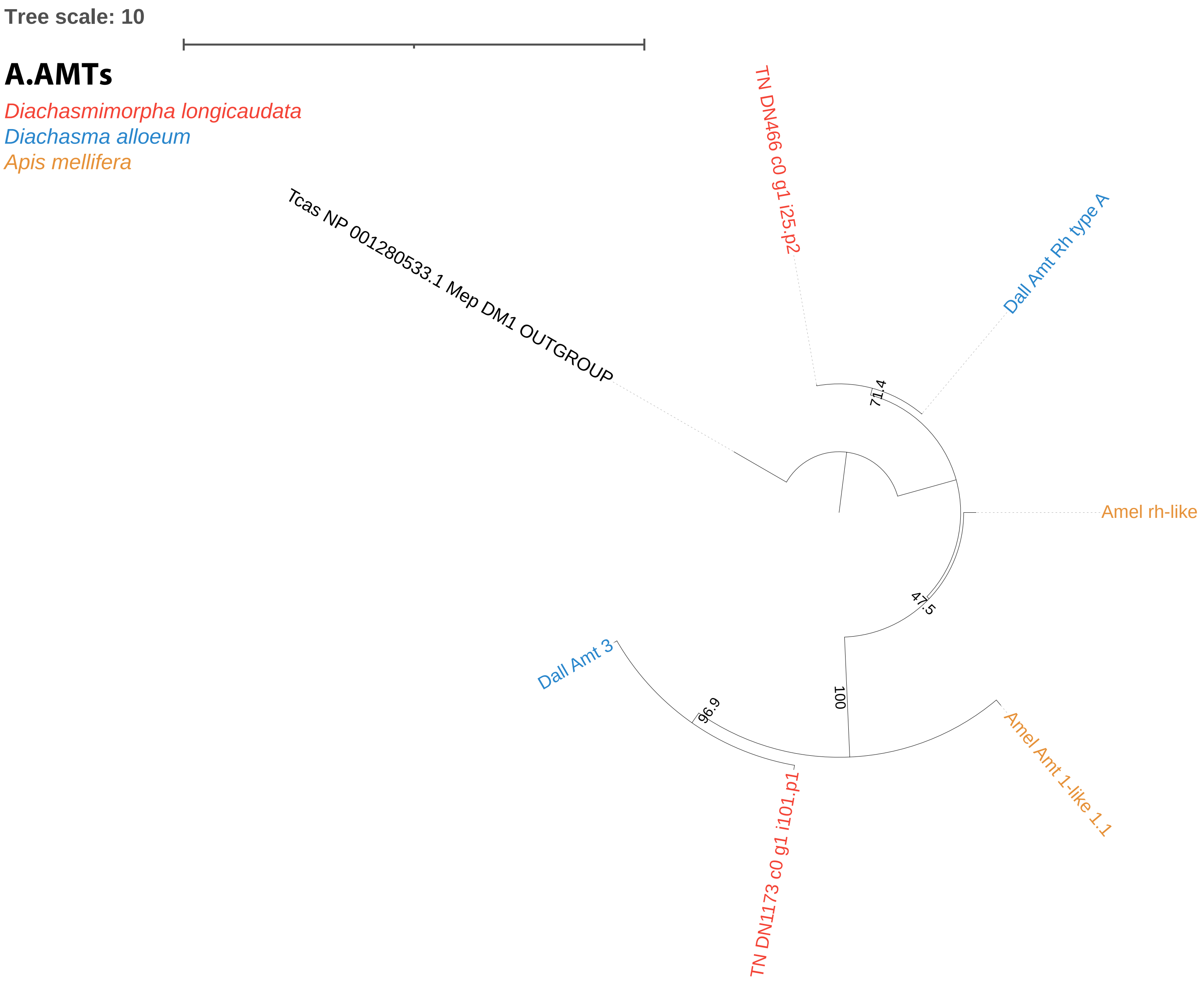

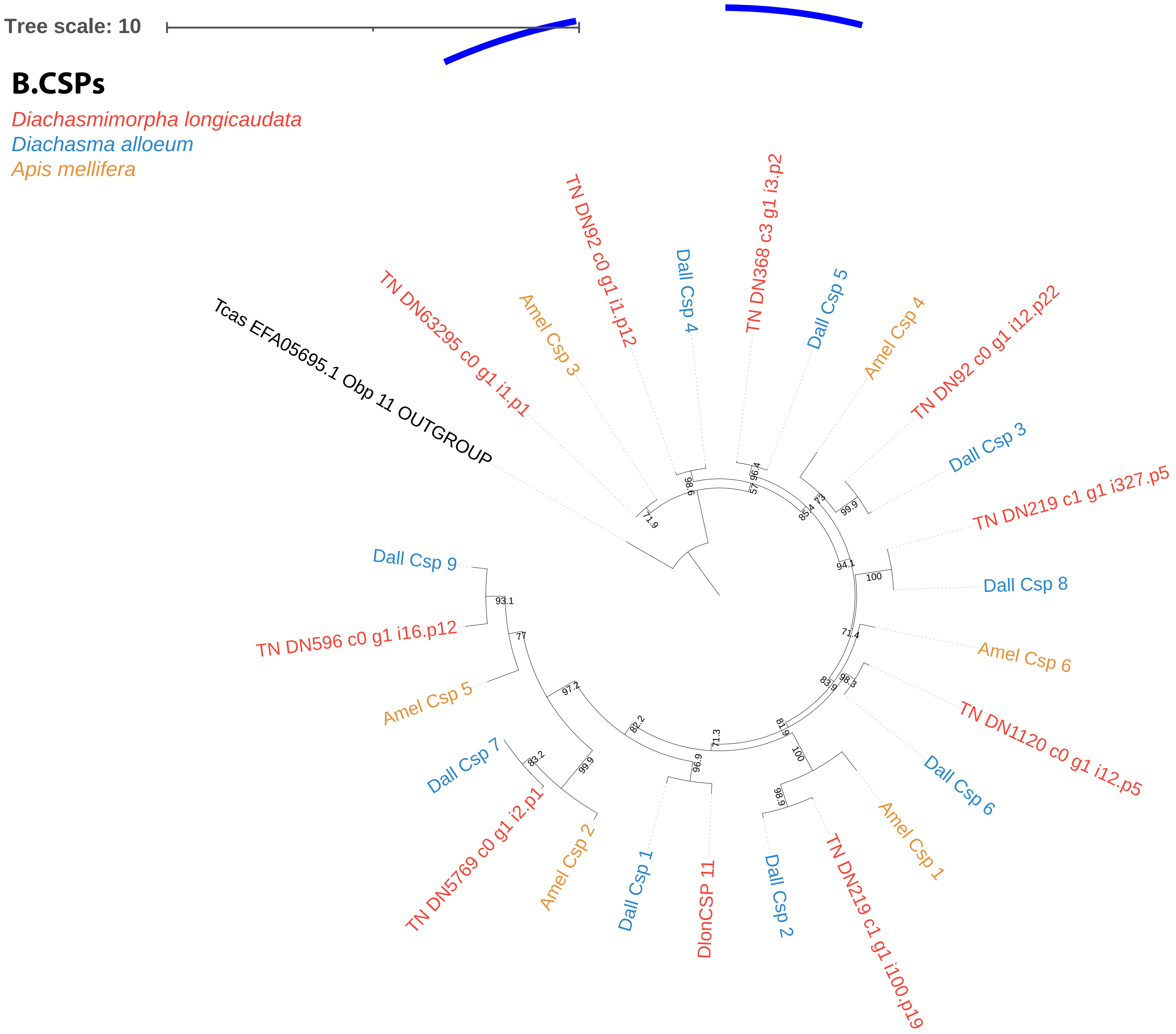
Tree scale: 10
B.CSPs
Diachasmimorpha longicaudata
Diachasma alloeum
Apis mellifera
Tcas EFA05695.1 Obp 11 OUTGROUP
TN DN63295 c0 g1 i1.p1
Amel Csp 3
TN DN92 c0 g1 i1.p12
Dall Csp 4
TN DN368 c3 g1 i3.p2
Dall Csp 5
Amel Csp 4
TN DN92 c0 g1 i12.p22
Dall Csp 3
TN DN219 c1 g1 i327.p5
Dall Csp 8
Amel Csp 6
TN DN1120 c0 g1 i12.p5
Dall Csp 6
Amel Csp 1
TN DN219 c1 g1 i100.p19
Dall Csp 2
DlonCSP 11
Dall Csp 1
Amel Csp 2
TN DN5769 c0 g1 i2.p1
Dall Csp 7
Amel Csp 5
TN DN596 c0 g1 i16.p12
Dall Csp 9

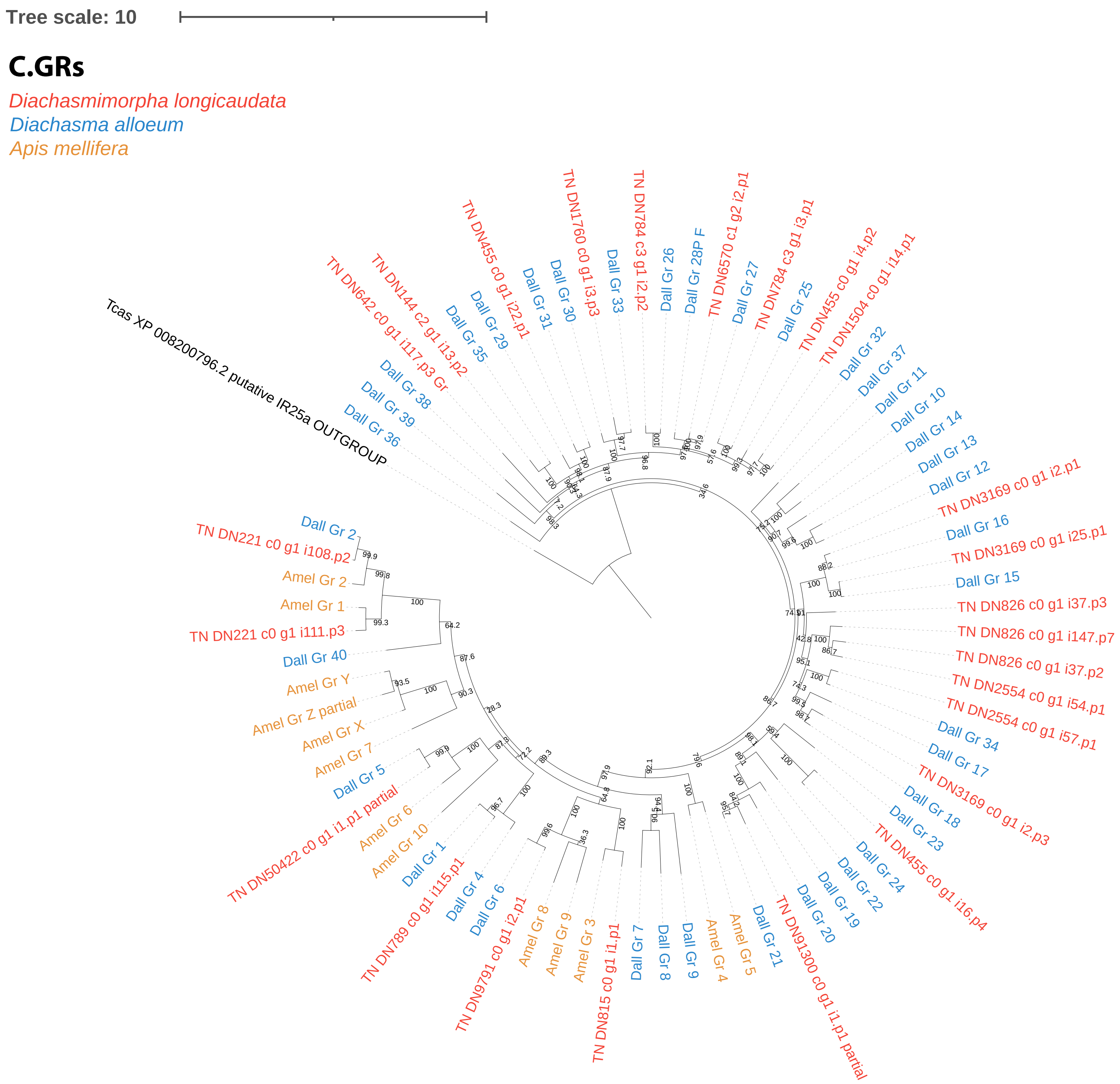
Tree scale: 10
C.GRs
Diachasmimorpha longicaudata
Diachasma alloeum
Apis mellifera
Tcas XP 008200796.2 putative IR25a OUTGROUP
Dall Gr 36
Dall Gr 39
Dall Gr 38
TN DN642 c0 g1 i117.p3 Gr
TN DN144 c2 g1 i13.p2
Dall Gr 35
Dall Gr 29
TN DN455 c0 g1 i22.p1
Dall Gr 31
Dall Gr 30
TN DN1760 c0 g1 i3.p3
Dall Gr 33
TN DN784 c3 g1 i2.p2
Dall Gr 26
Dall Gr 28P F
TN DN6570 c1 g2 i2.p1
Dall Gr 27
TN DN784 c3 g1 i3.p1
Dall Gr 25
TN DN455 c0 g1 i4.p2
TN DN1504 c0 g1 i14.p1
Dall Gr 32
Dall Gr 37
Dall Gr 11
Dall Gr 10
Dall Gr 14
Dall Gr 13
Dall Gr 12
TN DN3169 c0 g1 i2.p1
Dall Gr 16
TN DN3169 c0 g1 i25.p1
Dall Gr 15
TN DN826 c0 g1 i37.p3
TN DN826 c0 g1 i147.p7
TN DN826 c0 g1 i37.p2
TN DN2554 c0 g1 i54.p1
TN DN2554 c0 g1 i57.p1
Dall Gr 34
Dall Gr 17
TN DN3169 c0 g1 i2.p3
Dall Gr 18
Dall Gr 23
TN DN455 c0 g1 i16.p4
Dall Gr 24
Dall Gr 22
Dall Gr 19
Dall Gr 20
TN DN91300 c0 g1 i1.p1 partial
Dall Gr 21
Amel Gr 5
Amel Gr 4
Dall Gr 9
Dall Gr 8
Dall Gr 7
TN DN815 c0 g1 i1.p1
Amel Gr 3
Amel Gr 9
Amel Gr 8
TN DN9791 c0 g1 i2.p1
Dall Gr 6
Dall Gr 4
TN DN789 c0 g1 i115.p1
Dall Gr 1
Amel Gr 10
Amel Gr 6
TN DN50422 c0 g1 i1.p1 partial
Dall Gr 5
Amel Gr 7
Amel Gr X
Amel Gr Z partial
Amel Gr Y
Dall Gr 40
TN DN221 c0 g1 i111.p3
Amel Gr 1
Amel Gr 2
TN DN221 c0 g1 i108.p2
Dall Gr 2

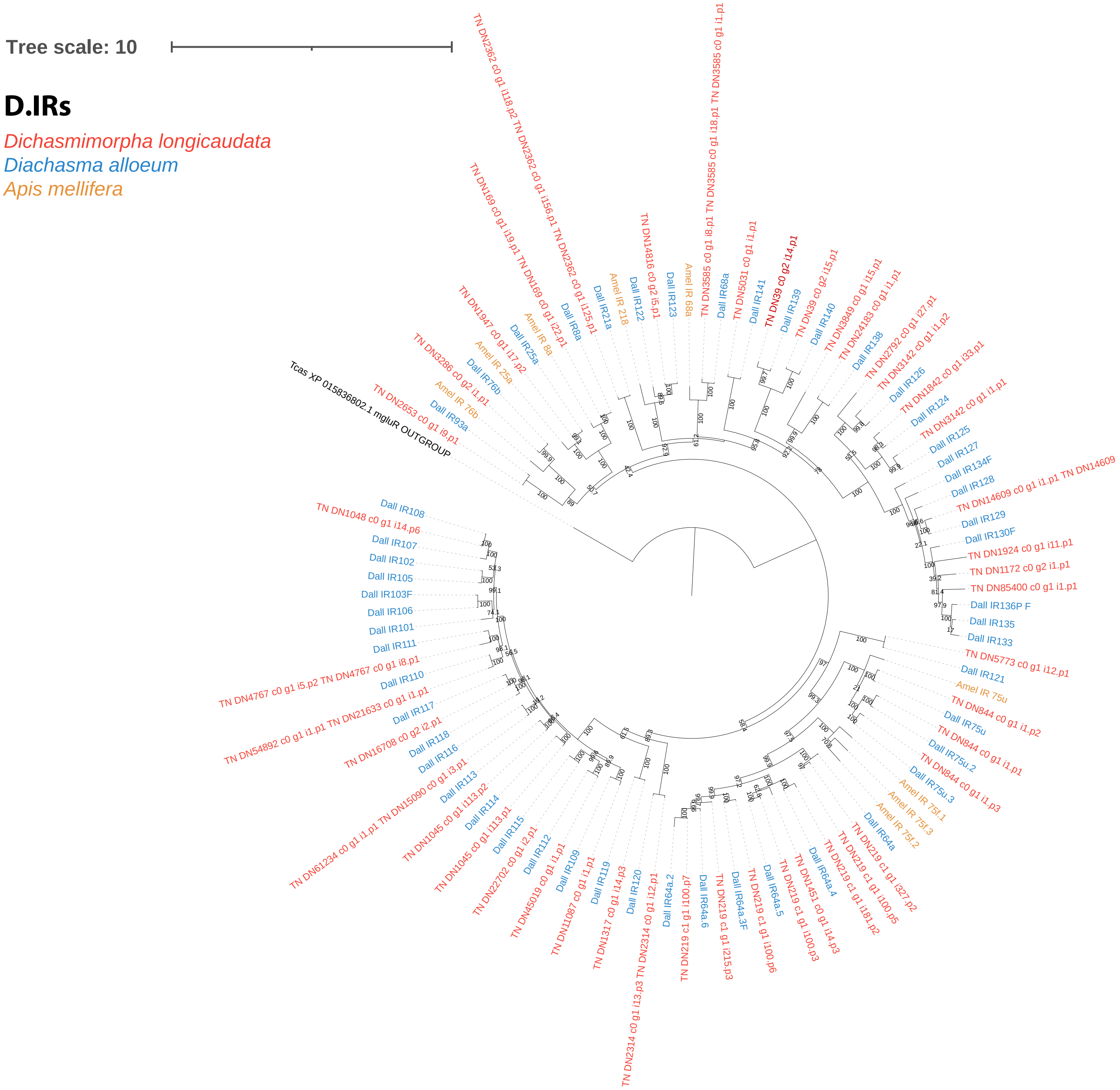
Tree scale: 10
D.IRs
Dichasmimorpha longicaudata
Diachasma alloeum
Apis mellifera
Tcas XP 015836802.1 mgluR OUTGROUP
TN DN2653 c0 g1 i9.p1
Dall IR93a
Amel IR 76b
TN DN3286 c0 g2 i1.p1
Dall IR76b
Amel IR 25a
TN DN1947 c0 g1 i17.p2
Dall IR25a
Amel IR 8a
TN DN169 c0 g1 i19.p1 TN DN169 c0 g1 i22.p1
Dall IR8a
TN DN2362 c0 g1 i118.p2 TN DN2362 c0 g1 i156.p1 TN DN2362 c0 g1 i125.p1
Dall IR21a
Amel IR 218
Dall IR122
TN DN14816 c0 g2 i5.p1
Dall IR123
Amel IR 68a
TN DN3585 c0 g1 i8.p1 TN DN3585 c0 g1 i18.p1 TN DN3585 c0 g1 i1.p1
Dall IR68a
TN DN5031 c0 g1 i1.p1
Dall IR141
TN DN39 c0 g2 i14.p1
Dall IR139
TN DN39 c0 g2 i15.p1
Dall IR140
TN DN3849 c0 g1 i15.p1
TN DN24183 c0 g1 i1.p1
Dall IR138
TN DN2792 c0 g1 i27.p1
TN DN3142 c0 g1 i1.p2
Dall IR126
TN DN1842 c0 g1 i33.p1
Dall IR124
TN DN3142 c0 g1 i1.p1
Dall IR125
Dall IR127
Dall IR134F
Dall IR128
TN DN14609 c0 g1 i1.p1 TN DN14609
Dall IR129
Dall IR130F
TN DN1924 c0 g1 i11.p1
TN DN1172 c0 g2 i1.p1
TN DN85400 c0 g1 i1.p1
Dall IR136P F
Dall IR135
Dall IR133
TN DN5773 c0 g1 i12.p1
Dall IR121
Amel IR 75u
TN DN844 c0 g1 i1.p2
Dall IR75u
TN DN844 c0 g1 i1.p1
Dall IR75u.2
TN DN844 c0 g1 i1.p3
Dall IR75u.3
Amel IR 75f.1
Amel IR 75f.3
Amel IR 75f.2
Dall IR64a
TN DN219 c1 g1 i327.p2
TN DN219 c1 g1 i100.p5
TN DN219 c1 g1 i181.p2
Dall IR64a.4
TN DN1451 c0 g1 i14.p3
TN DN219 c1 g1 i100.p3
Dall IR64a.5
TN DN219 c1 g1 i100.p6
Dall IR64a.3F
TN DN219 c1 g1 i215.p3
Dall IR64a.6
TN DN219 c1 g1 i100.p7
Dall IR64a.2
TN DN2314 c0 g1 i13.p3 TN DN2314 c0 g1 i12.p1
Dall IR120
TN DN1317 c0 g1 i14.p3
Dall IR119
TN DN11087 c0 g1 i1.p1
Dall IR109
TN DN45019 c0 g1 i1.p1
Dall IR112
TN DN22702 c0 g1 i2.p1
Dall IR115
TN DN1045 c0 g1 i113.p1
Dall IR114
TN DN1045 c0 g1 i113.p2
Dall IR113
TN DN61234 c0 g1 i1.p1 TN DN15090 c0 g1 i3.p1
Dall IR116
Dall IR118
TN DN16708 c0 g2 i2.p1
Dall IR117
TN DN54892 c0 g1 i1.p1 TN DN21633 c0 g1 i1.p1
Dall IR110
TN DN4767 c0 g1 i5.p2 TN DN4767 c0 g1 i8.p1
Dall IR111
Dall IR101
Dall IR106
Dall IR103F
Dall IR105
Dall IR102
Dall IR107
TN DN1048 c0 g1 i14.p6
Dall IR108

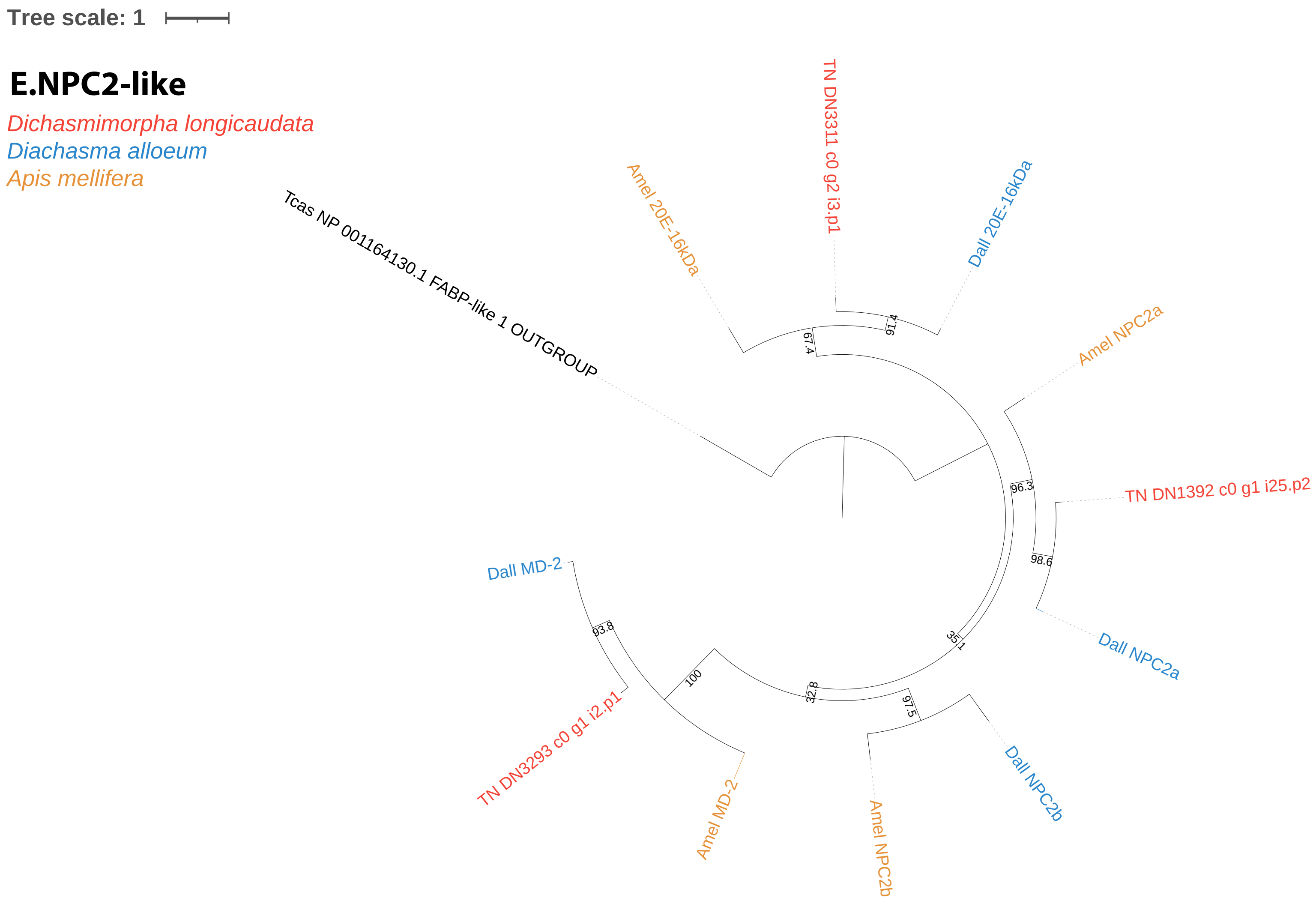

Tree scale: 1
E.NPC2-like
Dichasmimorpha longicaudata
Diachasma alloeum
Apis mellifera
Tcas NP 001164130.1 FABP-like 1 OUTGROUP
Amel 20E-16kDa
TN DN3311 c0 g2 i3.p1
Dall 20E-16kDa
Amel NPC2a
TN DN1392 c0 g1 i25.p2
Dall NPC2a
Dall NPC2b
Amel NPC2b
Amel MD-2
TN DN3293 c0 g1 i2.p1
Dall MD-2
67.4
91.4
96.3
98.6
35.1
32.8
97.5
100
93.8

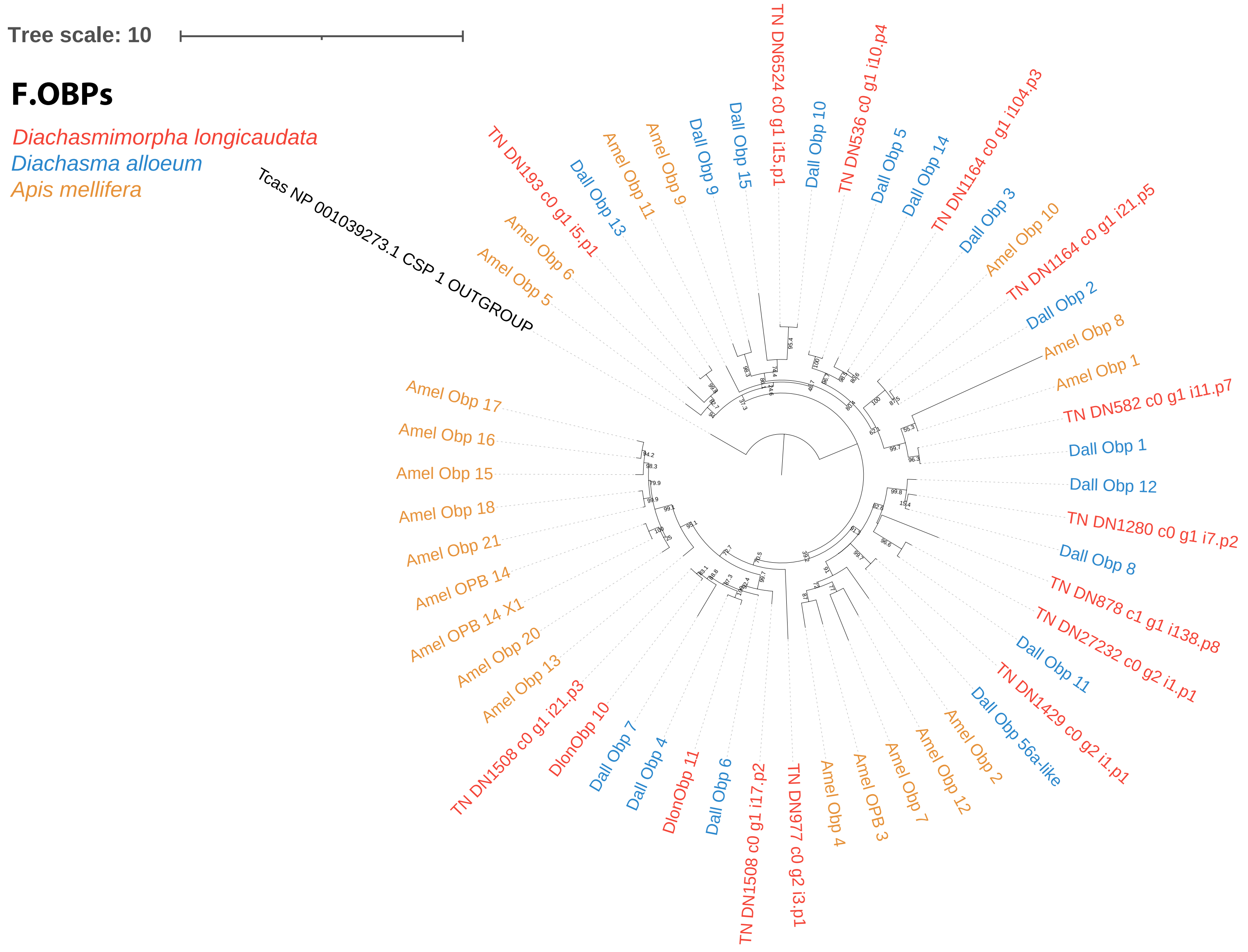

Tree scale: 10
F.OBPs
Diachasmimorpha longicaudata
Diachasma alloeum
Apis mellifera
Tcas NP 001039273.1 CSP 1 OUTGROUP
Amel Obp 5
Amel Obp 6
TN DN193 c0 g1 i5.p1
Dall Obp 13
Amel Obp 11
Amel Obp 9
Dall Obp 9
Dall Obp 15
TN DN6524 c0 g1 i15.p1
Dall Obp 10
TN DN536 c0 g1 i10.p4
Dall Obp 5
Dall Obp 14
TN DN1164 c0 g1 i104.p3
Dall Obp 3
Amel Obp 10
TN DN1164 c0 g1 i21.p5
Dall Obp 2
Amel Obp 8
Amel Obp 1
TN DN582 c0 g1 i11.p7
Dall Obp 1
Dall Obp 12
TN DN1280 c0 g1 i7.p2
Dall Obp 8
TN DN878 c1 g1 i138.p8
TN DN27232 c0 g2 i1.p1
Dall Obp 11
TN DN1429 c0 g2 i1.p1
Dall Obp 56a-like
Amel Obp 2
Amel Obp 12
Amel Obp 7
Amel OPB 3
Amel Obp 4
TN DN977 c0 g2 i3.p1
TN DN1508 c0 g1 i17.p2
Dall Obp 6
DlonObp 11
Dall Obp 4
Dall Obp 7
DlonObp 10
TN DN1508 c0 g1 i21.p3
Amel Obp 13
Amel Obp 20
Amel OPB 14 X1
Amel OPB 14
Amel Obp 21
Amel Obp 18
Amel Obp 15
Amel Obp 16
Amel Obp 17

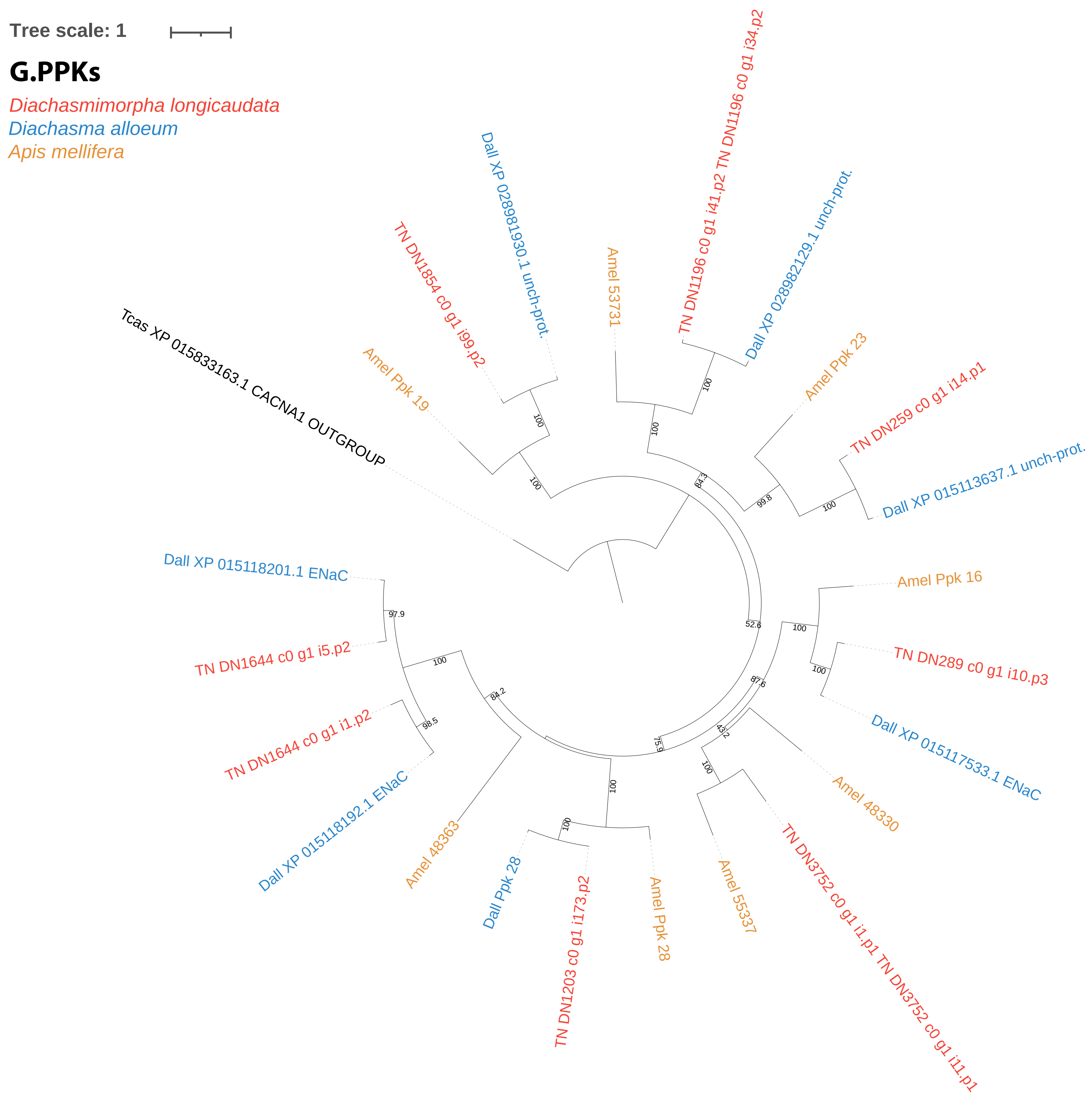

Tree scale: 1
G.PPKs
Diachasmimorpha longicaudata
Diachasma alloeum
Apis mellifera
Tcas XP 015833163.1 CACNA1 OUTGROUP
Amel Ppk 19
TN DN1854 c0 g1 i99.p2
Dall XP 028981930.1 unch-prot.
Amel 53731
TN DN1196 c0 g1 i41.p2 TN DN1196 c0 g1 i34.p2
Dall XP 028982129.1 unch-prot.
Amel Ppk 23
TN DN259 c0 g1 i14.p1
Dall XP 015113637.1 unch-prot.
Amel Ppk 16
TN DN289 c0 g1 i10.p3
Dall XP 015117533.1 ENaC
Amel 48330
TN DN3752 c0 g1 i1.p1 TN DN3752 c0 g1 i11.p1
Amel 55337
Amel Ppk 28
TN DN1203 c0 g1 i173.p2
Dall Ppk 28
Amel 48363
Dall XP 015118192.1 ENaC
TN DN1644 c0 g1 i1.p2
TN DN1644 c0 g1 i5.p2
Dall XP 015118201.1 ENaC
100
100
100
100
84.3
99.8
100
52.6
100
100
87.6
43.2
75.9
100
100
100
84.2
100
97.9
98.5

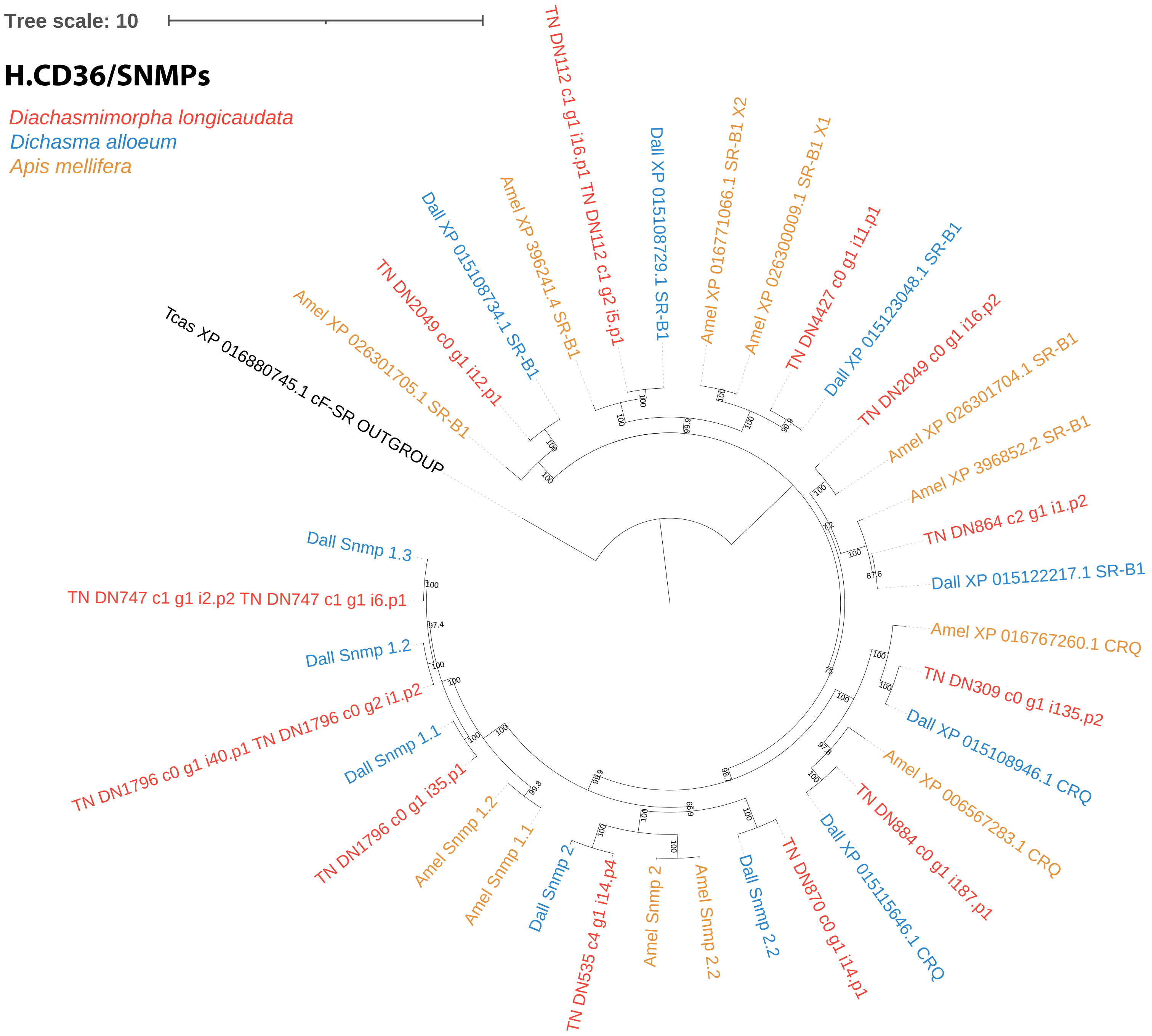

Tree scale: 10
H.CD36/SNMPs
Diachasmimorpha longicaudata
Dichasma alloeum
Apis mellifera
Tcas XP 016880745.1 cF-SR OUTGROUP
Amel XP 026301705.1 SR-B1
TN DN2049 c0 g1 i12.p1
Dall XP 015108734.1 SR-B1
Amel XP 396241.4 SR-B1
TN DN112 c1 g1 i16.p1 TN DN112 c1 g2 i5.p1
Dall XP 015108729.1 SR-B1
Amel XP 016771066.1 SR-B1 X2
Amel XP 026300009.1 SR-B1 X1
TN DN4427 c0 g1 i11.p1
Dall XP 015123048.1 SR-B1
TN DN2049 c0 g1 i16.p2
Amel XP 026301704.1 SR-B1
Amel XP 396852.2 SR-B1
TN DN864 c2 g1 i1.p2
Dall XP 015122217.1 SR-B1
Amel XP 016767260.1 CRQ
TN DN309 c0 g1 i135.p2
Dall XP 015108946.1 CRQ
Amel XP 006567283.1 CRQ
TN DN884 c0 g1 i187.p1
Dall XP 015115646.1 CRQ
TN DN870 c0 g1 i14.p1
Dall Snmp 2.2
Amel Snmp 2.2
Amel Snmp 2
TN DN535 c4 g1 i14.p4
Dall Snmp 2
Amel Snmp 1.1
Amel Snmp 1.2
TN DN1796 c0 g1 i35.p1
Dall Snmp 1.1
TN DN1796 c0 g1 i40.p1 TN DN1796 c0 g2 i1.p2
Dall Snmp 1.2
TN DN747 c1 g1 i2.p2 TN DN747 c1 g1 i6.p1
Dall Snmp 1.3

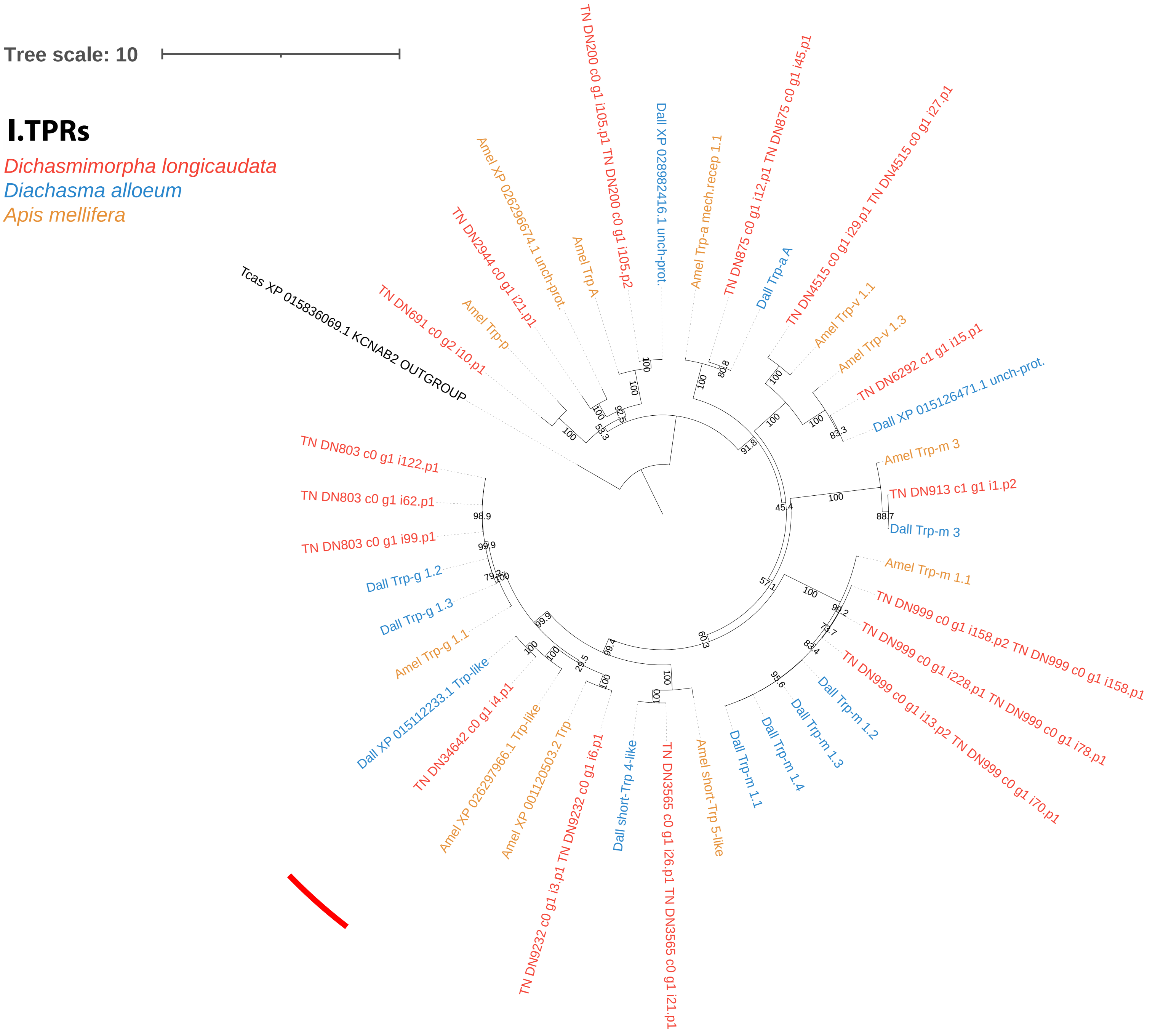

Tree scale: 10
I.TPRs
Dichasmimorpha longicaudata
Diachasma alloeum
Apis mellifera
Tcas XP 015836069.1 KCNAB2 OUTGROUP
TN DN691 c0 g2 i10.p1
Amel Trp-p
TN DN2944 c0 g1 i21.p1
Amel XP 026296674.1 unch-prot.
Amel Trp A
TN DN200 c0 g1 i105.p1 TN DN200 c0 g1 i105.p2
Dall XP 028982416.1 unch-prot.
Amel Trp-a mech.recep 1.1
TN DN875 c0 g1 i12.p1 TN DN875 c0 g1 i45.p1
Dall Trp-a A
TN DN4515 c0 g1 i29.p1 TN DN4515 c0 g1 i27.p1
Amel Trp-v 1.1
Amel Trp-v 1.3
TN DN6292 c1 g1 i15.p1
Dall XP 015126471.1 unch-prot.
Amel Trp-m 3
TN DN913 c1 g1 i1.p2
Dall Trp-m 3
Amel Trp-m 1.1
TN DN999 c0 g1 i158.p2 TN DN999 c0 g1 i158.p1
TN DN999 c0 g1 i228.p1 TN DN999 c0 g1 i78.p1
TN DN999 c0 g1 i13.p2 TN DN999 c0 g1 i70.p1
Dall Trp-m 1.2
Dall Trp-m 1.3
Dall Trp-m 1.4
Dall Trp-m 1.1
Amel short-Trp 5-like
TN DN3565 c0 g1 i26.p1 TN DN3565 c0 g1 i21.p1
Dall short-Trp 4-like
TN DN9232 c0 g1 i3.p1 TN DN9232 c0 g1 i6.p1
Amel XP 001120503.2 Trp
Amel XP 026297966.1 Trp-like
TN DN34642 c0 g1 i4.p1
Dall XP 015112233.1 Trp-like
Amel Trp-g 1.1
Dall Trp-g 1.3
Dall Trp-g 1.2
TN DN803 c0 g1 i99.p1
TN DN803 c0 g1 i62.p1
TN DN803 c0 g1 i122.p1
