# Supplementary material for: Identification of candidate genes associated with host-seeking behavior in the parasitoid wasp Diachasmimorpha longicaudata
Source: BMC Genomics. 2024 Feb 6;25:147. doi: 10.1186/s12864-024-10034-6 (PMC10848486; doi:10.1186/s12864-024-10034-6)
Supplement: Supplementary file 7 — Supplementary Material 7 [file 12864_2024_10034_MOESM7_ESM.pdf]

Additional File 7: Supplementary Figure S3. Antennal expression of differentially expressed sequences between females and males of *Diachasmimorpha longicaudata*. A heatmap was plotted using a z-score calculated from the transformed count data, by means of a color scale in which blue/red represent the lowest/highest expression. The dendrogram on the left represents the results of the row hierarchical clustering. Abbreviations: MA: male antenna, FE: female antenna.

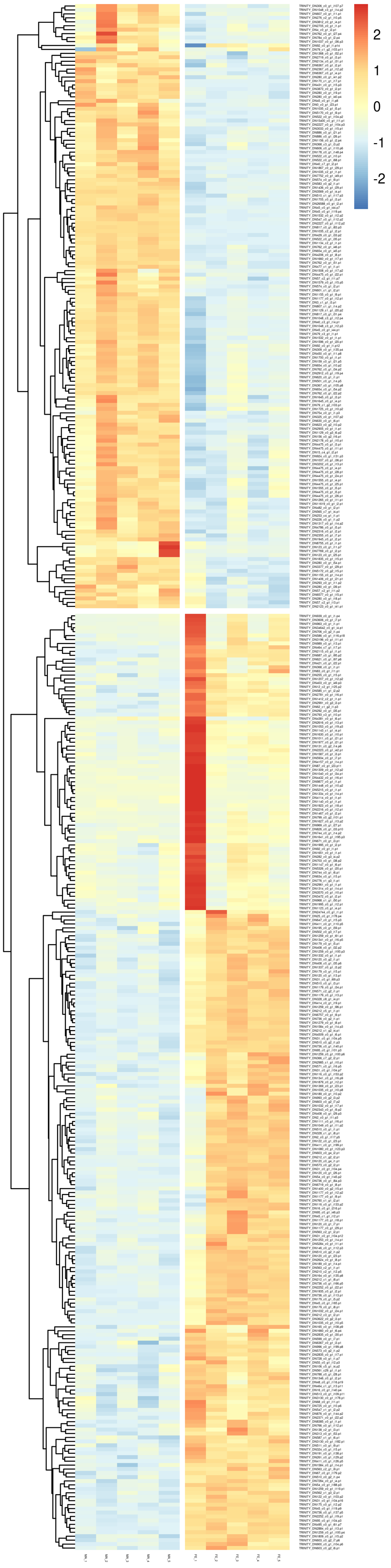
2
1
0
-1
-2
